# Nutraceutical Characterization, 3D Airway Tissue Metabolic Viability, and Cytotoxic Activity of *Melissa officinalis* Aqueous Leaf Extract in A549 Lung Adenocarcinoma Cells

**DOI:** 10.3390/medicina62081514

**Published:** 2026-08-06

**Authors:** Ioan-Alexandru Cîmpeanu, Alina Anton, Andreea-Maria Cristea, Diana Haj Ali, Iasmina-Alexandra Predescu, Iasmina Marcovici, Ioana-Gabriela Macaşoi, Daliborca Vlad, Cristian Oancea, Elena-Alina Moacă

**Affiliations:** 1 Doctoral School, Faculty of Medicine, “Victor Babeș” University of Medicine and Pharmacy, 2nd Eftimie Murgu Square, 300041 Timisoara, Romania; ioan.cimpeanu@umft.ro; 2 University Clinic of Toxicology, Drug Industry, Management, Marketing and Dermatopharmacy, Faculty of Pharmacy, “Victor Babes” University of Medicine and Pharmacy, 2nd Eftimie Murgu Square, 300041 Timisoara, Romania; dolghi.alina@umft.ro (A.A.); andreea.cristea@umft.ro (A.-M.C.); iasmina.marcovici@umft.ro (I.M.); macasoi.ioana@umft.ro (I.-G.M.); alina.moaca@umft.ro (E.-A.M.); 3 Research Centre for Pharmaco-Toxicological Evaluation, Faculty of Pharmacy, “Victor Babeș” University of Medicine and Pharmacy, 2nd Eftimie Murgu Square, 300041 Timisoara, Romania; 4 Discipline of Pharmacology, Department of Biochemistry and Pharmacology, Faculty of Medicine, “Victor Babeş” University of Medicine and Pharmacy, 2nd Eftimie Murgu Square, 300041 Timisoara, Romania; vlad.daliborca@umft.ro; 5 Discipline of Pneumology, Department of Infectious Diseases, Faculty of Medicine, “Victor Babes” University of Medicine and Pharmacy, 2nd Eftimie Murgu Square, 300041 Timisoara, Romania; oancea@umft.ro

**Keywords:** *Melissa*, plant extracts, antioxidants, polyphenols, lung adenocarcinoma, cell death, mitochondrial membrane potential, cell survival, cell culture techniques

## Abstract

*Background and Objectives*: *Melissa officinalis* (lemon balm) contains phenolic constituents with antioxidant and cytotoxic activities; however, evidence regarding aqueous leaf extracts in lung adenocarcinoma models and reconstructed human airway tissues remains limited. The novelty of the present study lies in the integrated evaluation of the same aqueous extract through phytochemical and antioxidant characterization, a three-dimensional reconstructed tracheobronchial tissue model, and complementary short- and long-term biological endpoints in A549 lung adenocarcinoma cells. *Materials and Methods*: Antioxidant activity was evaluated using the DPPH assay, total phenolic content (TPC) using the Folin–Ciocalteu method, and the targeted phenolic profile using UHPLC–MS/MS. EpiAirway^TM^ tissues were exposed to 1000 µg/mL extract for 24 h, and tissue metabolic viability was assessed using the MTT assay. A549 cells were treated with 20–1000 µg/mL extract for 24 h and evaluated using complementary metabolic, lysosomal, mitochondrial, morphological, membrane-integrity, and clonogenic endpoints. *Results*: The extract exhibited concentration-dependent DPPH radical-scavenging activity, with an estimated IC_50_ of 1.37 mg/mL (95% CI: 1.00–1.88 mg/mL), and a TPC of 10.84 ± 0.03 mg GAE/g dry extract. The most abundant targeted analytes were the unresolved 3,5-dihydroxybenzoic acid/3,4-dihydroxybenzoic acid group (2.09 ± 0.60 µg/mL) and the p-coumaric acid/trans-p-coumaric acid group (1.31 ± 0.04 µg/mL). EpiAirway^TM^ tissue viability was 85.87% following exposure to 1000 µg/mL. In A549 cells, metabolic viability decreased to approximately 59% and 53% at 500 and 1000 µg/mL, respectively, whereas neutral red uptake declined to approximately 50% at 1000 µg/mL. Treatment was also associated with mitochondrial membrane depolarization, concentration-dependent cellular and nuclear alterations, apoptotic-like and necrotic staining patterns, and a reduction in clonogenic capacity to approximately 21% at 1000 µg/mL. *Conclusions*: The aqueous *M. officinalis* leaf extract exhibited measurable antioxidant activity, did not cause a marked acute reduction in EpiAirway^TM^ tissue metabolic viability under the single exposure condition tested, and produced concentration-dependent cytotoxic and sustained antiproliferative effects in A549 cells. These exploratory findings do not establish comprehensive respiratory safety, a specific cell-death mechanism, or tumor selectivity. Nevertheless, the integrated experimental approach supports further investigation using standardized multiple extract batches, matched malignant and non-malignant lung epithelial models, and quantitative molecular endpoints.

## 1. Introduction

Lung cancer remains a major cause of cancer-related mortality, with non-small-cell lung cancer (NSCLC) accounting for most cases and lung adenocarcinoma representing its most common histological subtype. In Europe, NSCLC is the focus of various treatment strategies, including complementary herbal approaches studied as adjuvants to standard therapy [1,2,3]. Nowadays, there are a multitude of treatment options for lung adenocarcinoma, including specific surgical procedures (the optimal standard treatment for early stages), various combinations of chemotherapy, targeted therapy, radiotherapy, and immunotherapy [4]. However, some barriers have been observed, highlighting the need for new alternatives. Among the limitations and barriers are treatment resistance (primary or acquired), increased toxicity of treatments, and cost and accessibility [5]. Considering these aspects, over the years, multiple studies have been conducted with the common goal of discovering new alternatives that can improve current treatments, reduce toxicity, and be more affordable and accessible. It has been shown that traditional herbal medicines can enhance the efficacy of chemotherapy and modulate treatment-related toxicity in advanced NSCLC [1,2,3]. Therefore, plant-derived preparations remain of interest as sources of pharmacologically active constituents, and several experimental studies have reported cytotoxic or antiproliferative effects in NSCLC models, including A549 cells [1,2,3,6,7,8,9,10]. Some plant-derived compounds have also shown additive or synergistic effects when combined with conventional chemotherapeutic agents in preclinical studies [1,2,7]. Nevertheless, such preclinical findings are highly dependent on extract composition, experimental conditions, and the cellular model employed and do not independently establish clinical efficacy or therapeutic selectivity. Multiple studies have focused on investigating the selective cytotoxic effects of certain plant extracts against A549 lung cancer cells, without affecting normal lung cells, a desirable feature for potential nutraceutical strategies [7,8,11].

*Melissa officinalis* (lemon balm) is a plant known for its traditional use in promoting health. Lemon balm (Lb) belongs to the Lamiaceae family, rich in phytochemical compounds (mainly phenolic compounds—rosmarinic acid), known for their biological activities, especially antioxidant, anti-inflammatory, and anticancer effects, along with traditional uses as a sedative, anxiolytic, carminative, digestive aid, neuroprotective, and cognitive/mood and sleep enhancer [12,13,14,15,16]. Previous studies have investigated the effects of lemon balm extracts in several tumor cell models, including A549 cells; however, the available evidence varies considerably according to the plant material, extraction solvent, chemical composition, exposure duration, and cellular model employed [17,18]. Moreover, previous studies have rarely integrated detailed phytochemical and antioxidant characterization with complementary short- and long-term biological endpoints in malignant lung cells and an assessment of acute metabolic viability in reconstructed human airway tissue.

Aqueous extraction is particularly relevant because it is compatible with traditional preparations, favors the recovery of hydrophilic phenolic constituents, and avoids residual organic solvents. More extensive evidence indicates that plant-derived extracts may exert antioxidant and antiproliferative effects on the A549 cell line and may act synergistically with conventional cancer therapies [3,6,7,8,10,19,20]. Nevertheless, evidence regarding the phytochemical composition, antioxidant potential, and biological activity of aqueous *M. officinalis* leaf extracts in lung adenocarcinoma remains limited. This knowledge gap provided the rationale for the present study. The novelty of the experimental design lies in evaluating the same aqueous *M. officinalis* leaf extract using targeted UHPLC–MS/MS profiling, DPPH radical-scavenging and total phenolic content assays, a three-dimensional reconstructed human tracheobronchial tissue model, and multiple complementary endpoints in A549 lung adenocarcinoma cells. The EpiAirway^TM^ and A549 models were used to address distinct experimental questions and were not treated as matched malignant and non-malignant counterparts; therefore, their comparison was not intended to establish tumor selectivity.

Therefore, the present study aimed to prepare and characterize an aqueous *M. officinalis* leaf extract by evaluating its DPPH radical-scavenging activity, total phenolic content, and targeted UHPLC–MS/MS phenolic profile. The study further evaluated metabolic viability in a three-dimensional (3D) reconstructed human tracheobronchial airway tissue model (EpiAirway^TM^) following a single 24 h exposure. It investigated the cytotoxic and sustained antiproliferative effects of the extract in A549 lung adenocarcinoma cells using complementary metabolic, lysosomal, mitochondrial, morphological, cell-death, and clonogenic endpoints.

## 2. Materials and Methods

### 2.1. Plant Material Collection and Preparation of the Aqueous Extract

Leaves of *Melissa officinalis* L. were collected at the beginning of the flowering stage, from cultivated plants grown in the Botanical Garden of the Faculty of Pharmacy, “Victor Babeș” University of Medicine and Pharmacy, Timișoara, Romania. The plant material was botanically authenticated by Prof. Dr. Diana-Simona Tchiakpe-Antal from the Discipline of Pharmaceutical Botany. A voucher specimen was deposited in the Faculty Herbarium under voucher number 125/2016.

The leaves were dried in a plant-drying oven at 35 °C until constant mass was achieved, they were and subsequently ground to obtain a homogeneous plant powder. The aqueous extract was prepared according to a previously reported method [18], with minor modifications. Briefly, 2.00 g of dried and ground leaves were mixed with 200 mL of ultrapure water obtained using a Milli-Q^®^ Integral Water Purification System (Merck Millipore, Darmstadt, Germany), corresponding to a plant material-to-solvent ratio of 1:100 (*w*/*v*). The mixture was incubated for 48 h in an ES-20/60 orbital shaker–incubator (Biosan, Riga, Latvia) at 25 °C and 250 rpm. Following orbital extraction, the mixture was sonicated for 60 min using a 750 W QSonica probe sonicator (QSonica, Newtown, CT, USA) operated at 50% amplitude and using a pulsed mode of 5 s on and 5 s off. After sonication, the mixture was allowed to stand for 30 min at room temperature to facilitate the sedimentation of suspended plant particles and was filtered through a Whatman No. 4 filter paper. The resulting filtrate was dried in a POL-EKO laboratory oven (POL-EKO Aparatura, Wodzisław Śląski, Poland) at 40 °C for 48 h. The dried extract was weighed, transferred to an airtight light-protected container, and stored at 4 °C for approximately 1 day before phytochemical characterization and biological testing. Residual moisture, water activity, and chemical or microbiological stability during storage were not evaluated.

A total of 268.8 mg of dried extract was recovered, corresponding to an extraction yield of 13.44% (*w*/*w*) relative to the initial dry plant material. A single independently prepared extraction batch was used for all subsequent phytochemical characterization and biological experiments.

**Figure 1:**
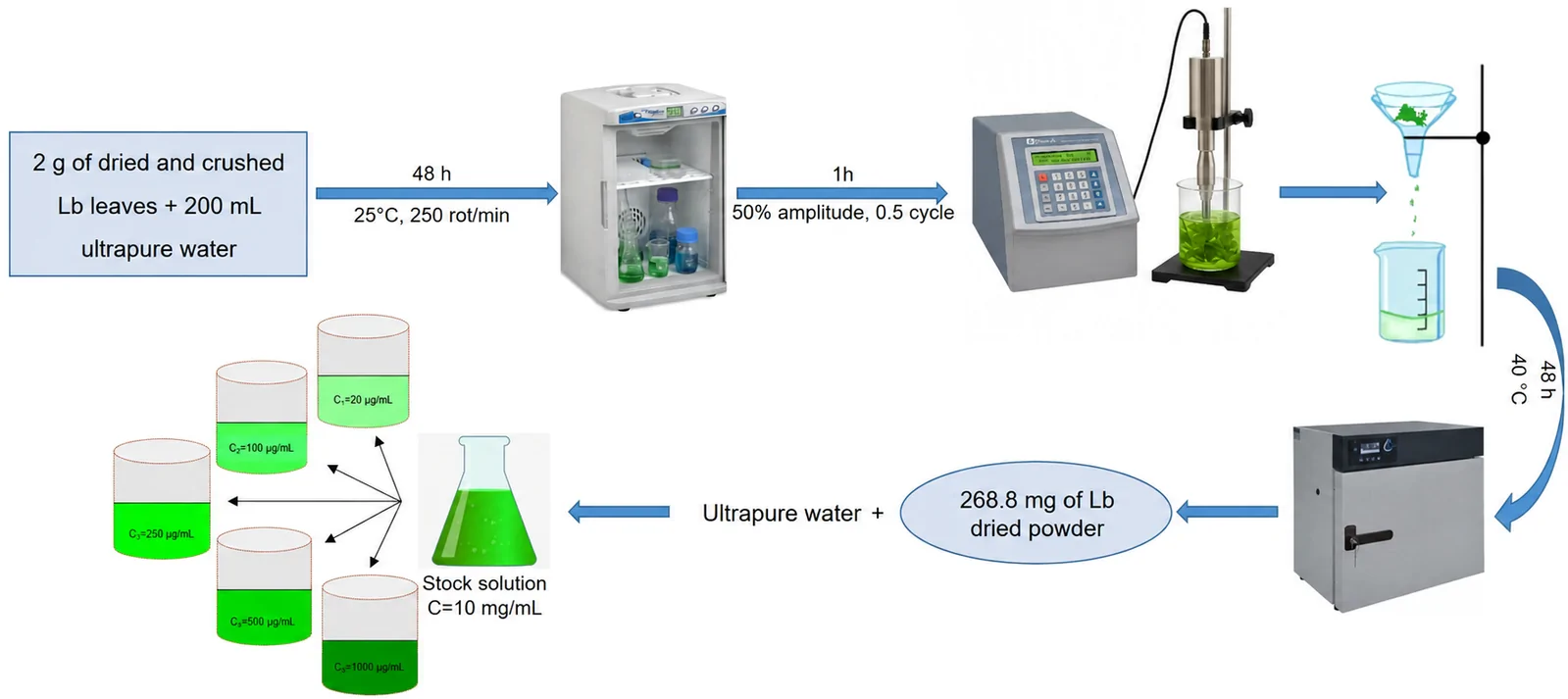
Schematic workflow for the preparation of the aqueous extract from dried *Melissa officinalis* leaves and the preparation of the stock and working solutions.

The dried extract was dissolved in ultrapure water to obtain a 10 mg/mL stock solution. The stock solution was subsequently diluted in the appropriate assay medium to obtain the concentrations required for each experiment. For the biological assessments, final extract concentrations of 20, 100, 250, 500, and 1000 µg/mL were prepared immediately before use. The preparation procedure is summarized in Figure 1.

### 2.2. Nutraceutical Characterization of Lb Aqueous Extract

#### 2.2.1. Antioxidant Capacity (AOC)

The AOC of the Lb aqueous extract at various concentrations (0.5 mg/mL, 1 mg/mL, 3 mg/mL, 5 mg/mL, and 10 mg/mL) was assessed using the DPPH (2,2-diphenyl-1-picrylhydrazyl) (purchased from Sigma-Aldrich, Steinheim, Germany) radical scavenging assay, following the protocol previously established by our group [21]. A 1 mM DPPH solution in ethanol was prepared and stored in the dark at 4 °C until analysis. The absorbance of the reaction mixtures was recorded at 516 nm with a T70 UV/VIS Spectrophotometer (from PG Instruments Ltd., Lutterworth, UK) until a stable reading was obtained. All determinations were compared with those obtained for an ethanolic solution of 0.4 mg/mL of ascorbic acid (acquired from Lach-Ner Company, Prague, Czech Republic), used as the reference standard.

The antioxidant capacity was obtained by applying the following formula:

$$AOC\left[\%\right]=\frac{A_{DPPH}-A_{sample}}{A_{DPPH}}\times 100$$

where AOC represents the antioxidant capacity [%]; $A_{DPPH}$ is the absorbance of the DPPH free radical, without Lb aqueous extract, recorded at 516 nm; $A_{sample}$ is the absorbance of the Lb aqueous extract mixed with the DPPH free radical.

All DPPH determinations were performed in triplicate on the same extract preparation, corresponding to three technical replicate measurements per concentration. The final DPPH radical-scavenging values recorded after stabilization of the reaction were averaged for each concentration and used to construct the concentration–response curve. The half-maximal inhibitory concentration (IC_50_) was estimated by nonlinear regression using GraphPad Prism software, version 11.0.2 (GraphPad Software, San Diego, CA, USA). Extract concentrations were log10-transformed and fitted using a normalized variable-slope sigmoidal model, with the Bottom and Top parameters constrained to 0% and 100%, respectively, according to the equation below. The IC_50_ estimate is reported together with its 95% confidence interval.

$$Y\;[\%]=\frac{100}{1+10^{(log{IC}_{50}-X)\times h}}$$

where Y represents the DPPH radical inhibition (%), X represents the logarithm of the extract concentration expressed in mg/mL, and h represents the Hill slope. The goodness of fit was evaluated using the coefficient of determination (R^2^).

#### 2.2.2. Total Phenolic Content (TPC)

The TPC of the Lb aqueous extract was determined using a slightly modified Folin–Ciocalteu colorimetric assay [22]. Gallic acid solutions prepared over the concentration range of 0.1–1 mg/mL were used to construct the calibration curve. An aliquot of 0.5 mL of the Lb aqueous extract solution (10 mg/mL) was mixed with 2.5 mL of Folin–Ciocalteu reagent diluted 1:10 with ultrapure water and 2 mL of 7.5% Na_2_CO_3_ solution. The reaction mixture was incubated for 2 h at room temperature in the dark. Absorbance was subsequently measured at 750 nm using a T70 UV/VIS spectrophotometer (PG Instruments Ltd.) against a blank [22,23]. TPC was calculated from the gallic acid calibration curve and expressed as mg gallic acid equivalents per gram of dry extract (mg GAE/g dry extract). All measurements were performed in triplicate, and the results are presented as mean ± standard deviation (SD).

#### 2.2.3. UHPLC–MS/MS Analysis of Phenolic Compounds

For the UHPLC analysis, solvents such as methanol, ultrapure water, and formic acid of LC–MS grade were purchased from VWR Chemicals (International Europe Services S.R.L., Bucharest, Romania). Native analytical standards and isotope-labeled standards were obtained from Dr. Ehrenstorfer (Augsburg, Germany) and Toronto Research Chemicals (Toronto, ON, Canada), as follows: trans-p-Coumaric acid (DRE-C11734100), Caffeic acid (DRE-C10934700), Rosmarinic acid (DRE-C16819500), Gallic acid (DRE-C13998280), trans-Ferulic acid (DRE-C13644100), (E/Z)-Ferulic acid (DRE-C13644090), 3,4-Dihydroxybenzoic acid (DRE-C12634710), Rutin (DRE-C16880000), Chlorogenic acid (DRE-C11415750), Quercetin (DRE-C16695000), Gallic acid D2 (DRE-C13998281), and Genistein (DRE-C13999800) from dr. Ehrenstorfer, and 3,5-Dihydroxybenzoic Acid (D451700), 2,4-Dihydroxybenzoic Acid (D678940), Resveratrol (R150000), p-Coumaric acid (C755365), trans-p-Coumaric-d6 Acid (C755392), Caffeic Acid-13C3 (C080002), Ferulic Acid-d3 (F308902), 3,4-Dihydroxybenzoic Acid-d3 (TRC-D451682), Rutin-d3 (R701800), Chlorogenic acid 13C3 (TRC-C366542), Quercetin-d3 (Q509502), Genistein d4 (G350010), and Resveratrol 13C6 (R150002) from Toronto Research Chemicals.

The phenolic profile of the Lb aqueous extract was investigated using a Shimadzu UHPLC system coupled to an LCMS-8045 triple-quadrupole mass spectrometer (Shimadzu Corporation, Kyoto, Japan), according to a previously validated method [24]. The chromatographic system consisted of two LC-30AD solvent-delivery pumps, a DGU-20A5R online degassing unit, a SIL-30AC autosampler, a CTO-20AC column oven, and a CBM-20A system controller, all manufactured by Shimadzu Corporation (Kyoto, Japan). Data acquisition and processing were performed using LabSolutions software, version 5.86.

A 5 µL aliquot was injected into the chromatographic system, and the separation was performed on a C18 reversed-phase column (2.1 × 100 mm, 2.7 µm) maintained at 40 °C. The mobile phases consisted of methanol containing 0.05% formic acid (mobile phase A) and ultrapure water containing 0.05% formic acid (mobile phase B). The chromatographic gradient was initiated at 10% A and maintained until 5 min, increased to 95% A by 8 min, and subsequently returned to 10% A for column re-equilibration until 14 min. The flow rate was 0.4 mL/min, and the total analytical run time was 14 min. To facilitate comparison with the total phenolic content and with literature data, analyte concentrations measured in the analytical solution were also normalized to the mass of dry extract. The concentrations expressed as mg/g dry extract were calculated according to the following relationship:

$$C_{analyte}\;\left(\frac{mg}{g}dry\;extract\right)=\;C_{analyte}\;\times \;\frac{DF}{C_{extract}}$$

where DF represents the applicable dilution factor, and C_extract_ represents the concentration of dry extract in the analyzed solution. For the 10 mg/mL extract solution analyzed in the present study, with no additional dilution factor applied, the measured concentrations were divided by 10.

Mass-spectrometric detection was performed using an electrospray ionization source and multiple-reaction-monitoring transitions. Analytes were identified based on their retention times and characteristic precursor-to-product ion transitions. Quantification was performed using isotope-labeled internal-standard-corrected calibration curves over the range of 50–5000 ng/mL (0.05–5.00 µg/mL), with coefficients of determination exceeding 0.998. The reported limit of detection and limit of quantification were 15 and 50 ng/mL, respectively. The UHPLC–MS/MS analysis was performed in three technical determinations using the same analytical solution prepared from the single extract batch. Results are expressed as mean ± standard deviation (SD). Accordingly, the reported SD represents within-sample analytical repeatability and does not reflect variability between independently prepared extract batches.

### 2.3. In Vitro Assessment

#### 2.3.1. Reagents and Instruments

The AIR-100-MM medium and TEER buffer were acquired from MatTek Life Sciences (Bratislava, Slovak Republic). Phosphate-buffered saline (PBS), dimethyl sulfoxide (DMSO), F-12K Medium (Kaighn’s Modification of Ham’s F-12 Medium), penicillin/streptomycin, and fetal bovine serum (FBS) were purchased from ATCC (Manassas, VA, USA). The MTT viability reagent [3-(4,5-dimethylthiazol-2-yl)-2,5-diphenyltetrazolium bromide] was supplied by Roche Holding AG (Basel, Switzerland). Hoechst 33342 and MitoTracker^TM^ Red CMXRos fluorescent dyes were obtained from Thermo Fisher Scientific (Waltham, MA, USA), whereas the JC-1 Mitochondrial Membrane Potential Assay Kit was acquired from Elabscience (Houston, TX, USA). Acridine Orange (AO), Propidium Iodide (PI), and sodium lauryl sulfate (SLS) were obtained from Sigma-Aldrich (Merck KGaA, Darmstadt, Germany). A 4% paraformaldehyde solution in PBS was purchased from Santa Cruz Biotechnology (Dallas, TX, USA), while the 1% crystal violet solution was obtained from Electron Microscopy Sciences (Hatfield, PA, USA).

Experimental data were collected using a Cytation 5 multimode microplate reader, while microscopic images were captured with a Lionheart FX Automated Microscope, both manufactured by BioTek Instruments (Winooski, VT, USA). The resulting datasets were processed and analyzed using Gen5^TM^ Microplate Data Collection and Analysis Software (Version 3.14; BioTek Instruments, USA).

#### 2.3.2. Experimental Design

EpiAirway^TM^ three-dimensional reconstructed human airway tissues, obtained from MatTek Life Sciences (Bratislava, Slovak Republic), were used to assess tissue metabolic viability following a single 24 h exposure to the Lb aqueous extract.

Cytotoxic effects were assessed using the human lung epithelial carcinoma cell line A549 (CRM-CCL-185^TM^), which was purchased from the American Type Culture Collection (ATCC, Manassas, VA, USA). Cells were cultured in F-12K Medium (Kaighn’s Modification of Ham’s F-12 Medium) supplemented with 10% fetal bovine serum (FBS) and 1% penicillin-streptomycin solution and maintained in a humidified incubator at 37 °C with 5% CO_2_. The stock solution of the tested extract was prepared in ultrapure distilled water. Working solutions were subsequently prepared in F-12K culture medium to obtain final concentrations of 20, 100, 250, 500, and 1000 μg/mL. The 10 mg/mL aqueous stock solution was diluted directly in complete F-12K culture medium. Accordingly, the final proportions of the aqueous stock solution were 0.2%, 1%, 2.5%, 5%, and 10% (*v*/*v*) for the 20, 100, 250, 500, and 1000 µg/mL treatment solutions, respectively. The corresponding proportions of complete F-12K medium were 99.8%, 99%, 97.5%, 95%, and 90%, respectively. Untreated control wells received complete F-12K culture medium without extract or additional ultrapure water. The pH and osmolality of the final extract-containing treatment solutions were not measured immediately before cell exposure, and no pH adjustment or neutralization was performed. A549 cells were treated with the tested concentrations for 24 h before further analyses.

#### 2.3.3. Assessment of Acute Metabolic Viability Using 3D EpiAirway^TM^ Tissues

To assess acute tissue metabolic viability under a single high-exposure condition, EpiAirway^TM^ tissues were exposed to the Lb aqueous extract at 1000 µg/mL for 24 h. Because the EpiAirway^TM^ experiment was intended as an initial screening assessment rather than as a comprehensive concentration–response evaluation, the highest concentration evaluated in A549 cells (1000 µg/mL) was selected as a single high-concentration condition. A 24 h exposure period was used to maintain temporal consistency with the A549 experiments, and tissue metabolic viability was assessed using the MTT assay. No additional concentrations, exposure durations, or complementary tissue endpoints were included in this preliminary experiment. The EpiAirway^TM^ experiments were performed in three independent biological experiments, each including three technical replicates. A volume of 50 µL of the extract solution was applied to the apical surface of each tissue insert. The 1000 µg/mL EpiAirway^TM^ treatment solution was prepared by diluting the 10 mg/mL aqueous extract stock solution 1:10 in AIR-100-MM, resulting in a final aqueous-stock fraction of 10% (*v*/*v*). All experimental procedures were conducted in accordance with the manufacturer’s instructions. Upon arrival, the tissue inserts were carefully removed from the agarose matrix, gently blotted with sterile gauze, and transferred into 6-well culture plates containing 1 mL of AIR-100-MM medium per well. The tissues were then allowed to equilibrate for 16–18 h under standard culture conditions (37 °C, 5% CO_2_). Following equilibration, the medium was replaced, and the apical surface of each insert was rinsed twice with TEER buffer. Subsequently, the tissues were exposed to Lb aqueous extract by applying 50 µL of the sample to each insert and incubating them for 24 h. After treatment, the tissues were washed with TEER buffer and transferred to a 24-well plate containing 300 µL of MTT solution per well, followed by a 90 min incubation period. Formazan extraction was performed by transferring the inserts to a separate 24-well plate filled with MTT extraction solution. Absorbance values were measured at 570 nm and 650 nm, and tissue viability was calculated using a previously reported equation [25].

#### 2.3.4. Cell Viability Evaluation Using the MTT Test

The cytotoxic effect was initially evaluated by measuring cell viability using the MTT colorimetric assay. For this purpose, A549 cells were seeded in 96-well culture plates at a density of 1 × 10^4^ cells per well. Once the cultures reached approximately 70% confluence, the cells were treated for 24 h with Lb aqueous extract (20–1000 μg/mL). After exposure, the medium was discarded and replaced with fresh culture medium containing 10 μL of MTT reagent per well. The plates were subsequently incubated for 3 h at 37 °C under standard culture conditions (5% CO_2_, humidified atmosphere). Upon incubation, 100 μL of solubilization buffer was added to each well to dissolve the formed formazan crystals, and the plates were kept at room temperature, protected from light, for an additional 30 min. The absorbance values were then measured at 570 nm and 630 nm using a Cytation 5 microplate reader [26].

#### 2.3.5. Microscopic Assessment of Cell Morphology

The morphology of A549 cells treated with Lb aqueous extract (20–1000 μg/mL) was evaluated 24 h after treatment under brightfield illumination using a Lionheart FX automated microscope. Images were processed and analyzed using Gen5^TM^ Microplate Data Collection and Analysis Software (version 3.14; BioTek Instruments Inc.).

#### 2.3.6. Neutral Red Uptake

A549 cells were cultured in F-12K medium supplemented with 10% FBS and seeded into flat-bottom 96-well plates at a density of 1 × 10^4^ cells per well. After allowing sufficient time for cell attachment, the cultures were treated with Lb aqueous extract at concentrations of 20–1000 μg/mL for 24 h. At the end of the exposure period, the treatment medium was removed and replaced with 100 μL of neutral red solution (40 μg/mL prepared in culture medium) to facilitate lysosomal uptake of the dye. The cells were then incubated with the neutral red solution for 2 h at 37 °C in a humidified atmosphere containing 5% CO_2_. Following incubation, the wells were carefully rinsed with 150 μL of PBS to eliminate excess dye, and color bright-field images were captured using a Lionheart FX automated microscope. To extract the incorporated dye, 150 μL of a decolorizing solution composed of approximately 50% ethanol, 49% ultrapure water, and 1% glacial acetic acid was added to each well. Absorbance was subsequently measured at 540 nm using a Cytation 5 microplate reader, while additional microscopic images were acquired using the Lionheart FX imaging platform. The assay was performed according to a previously reported protocol [27].

#### 2.3.7. Mitochondrial Membrane Potential (ΔΨm) Assay—JC-1 Staining

The mitochondrial membrane potential in A549 cells treated with Lb aqueous extract (20–1000 µg/mL) for 24 h was assessed using the JC-1 dye according to a previously described protocol [28]. The cells were seeded in 96-well black-walled plates with transparent bottoms at a density of 1 × 10^4^ cells per well and incubated until optimal confluence was reached. After 24 h of treatment, the cells were washed with PBS and stained with JC-1 dye (5 µM, 100 µL/well), followed by a 45 min incubation at 37 °C and 5% CO_2_. After incubation, the cells were washed with PBS, and representative fluorescence images were acquired using the Lionheart FX microscope. Quantitative fluorescence measurements were obtained directly using the Cytation 5 microplate reader. For each well, the JC-1 aggregate-to-monomer ratio was calculated by dividing the red fluorescence intensity measured at 590 nm by the green fluorescence intensity measured at 529 nm:

$${JC-1\;ratio\;}_{red/green}=\;\frac{F_{590}}{F_{529}}$$

The ratio obtained for each treatment group was subsequently normalized to the mean ratio of the untreated control group, which was set to 100%, according to the following equation:

$$Relative\;JC-1\;ratio\;\left(\%\right)=\;\frac{{JC-1\;ratio\;}_{treated}}{{JC-1\;ratio\;}_{control}}\;\times 100$$

No additional normalization to cell number or cell viability was performed.

#### 2.3.8. Analysis of Nuclear and Mitochondrial Aspects Using Fluorescence Staining

The effects of Lb aqueous extract at concentrations of 20–1000 μg/mL on the nuclear and mitochondrial morphology of A549 cells were investigated by fluorescence microscopy, using a Lionheart FX automated microscope. Cells were seeded into 96-well plates and allowed to proliferate until reaching approximately 70% confluence, after which they were exposed to the test samples for 24 h. For visualization of mitochondria, the cells were incubated with MitoTracker^TM^ Red CMXRos prepared in F-12K at a final concentration of 300 nM for 30 min. The cells were then rinsed and subsequently stained with Hoechst 33342 solution (1:2000 dilution in PBS) for 5–10 min to stain the nuclei. Excess dye was removed by three consecutive washes with PBS. Fluorescence images were then acquired using a Lionheart FX automated microscope. The experimental procedure followed a previously reported protocol, with minor adaptations [29].

#### 2.3.9. Acridine Orange/Propidium Iodide (AO/PI) Assay

To qualitatively assess plasma membrane integrity and nuclear staining patterns associated with viable cells and cells displaying apoptotic-like or necrotic features, A549 cells were examined using acridine orange/propidium iodide (AO/PI) double staining after 24 h of treatment. Cells were seeded in 12-well plates at a density of 1 × 10^5^ cells/well and cultured under standard conditions. Following treatment with Lb aqueous extract (20–1000 μg/mL), 100 μL of AO/PI staining solution containing 10 μg/mL acridine orange and 10 μg/mL propidium iodide was added to each well. After incubation for 10 min at room temperature in the dark, fluorescence images were acquired using a Lionheart FX automated microscope and analyzed using Gen5^TM^ Microplate Data Collection and Analysis software [30].

#### 2.3.10. Colony Formation Assay

The effect of Lb aqueous extract on the long-term proliferative potential of A549 cells was investigated using a colony formation assay adapted from a previously reported method [31]. Initially, cells were seeded in 96-well plates at a density of 100 cells per well and allowed to adhere to the culture surface. After attachment, the cells were exposed to Lb aqueous extract at concentrations of 20–1000 µg/mL for 24 h. Following treatment, the culture medium was replaced with fresh medium, which was subsequently renewed at regular intervals during a 10-day growth period to permit colony development. After the incubation period, the colonies were fixed with 4% paraformaldehyde for 10 min at room temperature and rinsed twice with phosphate-buffered saline (PBS). The fixed cells were then stained with a 0.2% crystal violet solution prepared in PBS for an additional 10 min. Excess stain was removed by two washes with distilled water. Colony images were captured using a Lionheart FX automated imaging system and analyzed with Gen5 Microplate Data Collection and Analysis Software. For quantitative assessment, the stained cells were lysed with 1% sodium lauryl sulfate (SLS), and absorbance was measured at 550 nm using a Cytation 5 multimode microplate reader.

#### 2.3.11. Statistical Analysis

Unless otherwise stated, A549 cell-based experiments were performed in three independent biological experiments, each including three technical replicates. EpiAirway^TM^ experiments were likewise performed in three independent biological experiments, each including three technical replicates. Technical replicates were averaged before statistical analysis, and each independent biological experiment was considered the experimental unit. DPPH and TPC measurements were performed in triplicate on the same extract preparation, whereas the UHPLC–MS/MS analysis consisted of three technical determinations performed using the same analytical solution prepared from the single extract batch. Data are presented as mean ± standard deviation (SD). The normality of the data distribution was evaluated using the Shapiro–Wilk test. The vehicle-treated control and the 1000 µg/mL extract-treated EpiAirway^TM^ groups were compared using an unpaired two-tailed *t*-test. A549 cell experiments involving multiple extract concentrations were analyzed using one-way analysis of variance (ANOVA), followed by Dunnett’s multiple comparisons test against the untreated control. Statistical analyses were performed using GraphPad Prism software, version 11.0.2 (GraphPad Software, San Diego, CA, USA). Differences were considered statistically significant at *p* < 0.05 and are indicated as follows: * *p* < 0.05, ** *p* < 0.01, *** *p* < 0.001, and **** *p* < 0.0001.

## 3. Results

### 3.1. Nutraceutical Screening of Lb Aqueous Extract

#### 3.1.1. AOC Screening

**Figure 2:**
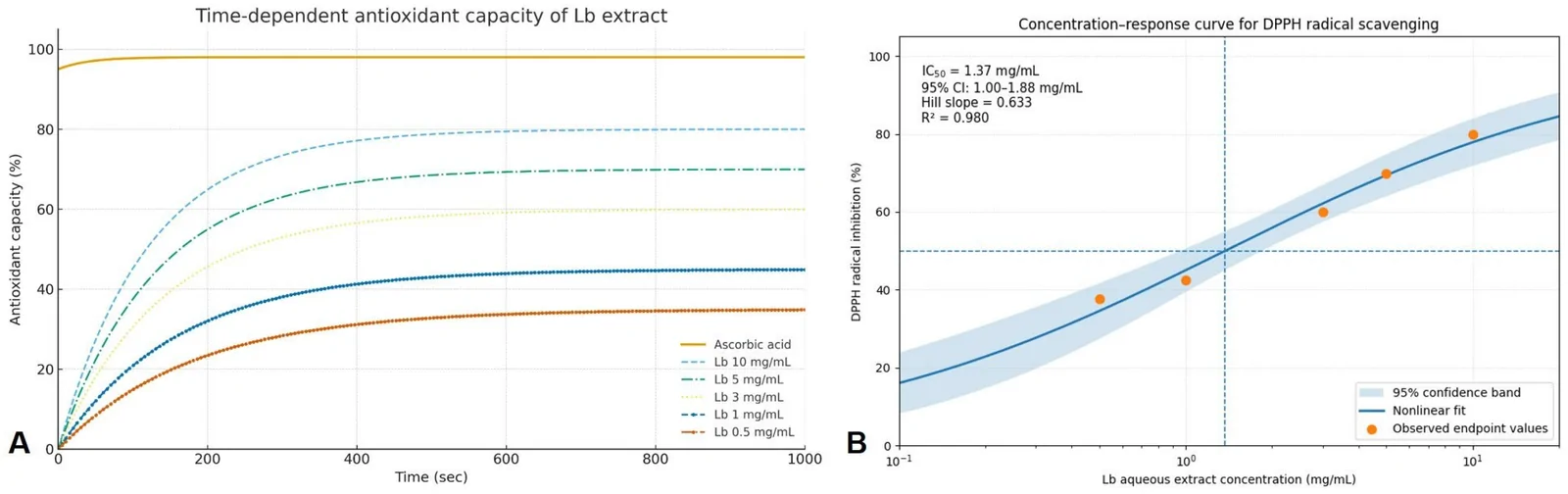
DPPH radical-scavenging activity of the Lb aqueous extract. (**A**) Time-dependent antioxidant activity of the extract at concentrations ranging from 0.5 to 10 mg/mL, compared with 0.4 mg/mL ascorbic acid. (**B**) Concentration–response curve constructed from the mean final stabilized inhibition values obtained from three technical replicate determinations per concentration. The solid line represents the nonlinear regression fit, and the shaded area represents its pointwise 95% confidence band. The IC_50_ was estimated using a normalized variable-slope sigmoidal model with the Bottom and Top constrained to 0% and 100%, respectively. The estimated IC_50_ was 1.37 mg/mL (95% CI: 1.00–1.88 mg/mL), with a Hill slope of 0.633 and R^2^ = 0.980.

Figure 2A illustrates the time-dependent DPPH radical-scavenging activity of the Lb aqueous extract at concentrations ranging from 0.5 to 10 mg/mL, compared with 0.4 mg/mL ascorbic acid used as the reference antioxidant. All tested concentrations exhibited measurable antioxidant activity, and the percentage of DPPH radical inhibition increased in a concentration-dependent manner. At the final stable measurement, the lowest tested concentration, 0.5 mg/mL, produced 37.56% inhibition, whereas 1, 3, and 5 mg/mL produced inhibition values of 42.38%, 60.02%, and 69.87%, respectively. The highest antioxidant activity was recorded for the 10 mg/mL extract, which inhibited 80.01% of the DPPH radicals.

The samples tested at 3, 5, and 10 mg/mL approached a stable response within approximately 500 s, whereas the lower concentrations of 0.5 and 1 mg/mL required approximately 700 s to reach equilibrium.

Nonlinear regression analysis of the concentration–response relationship yielded an estimated IC_50_ value of 1.37 mg/mL (95% CI: 1.00–1.88 mg/mL), corresponding to the concentration of Lb aqueous extract required to inhibit 50% of the DPPH radicals. The fitted curve had a Hill slope of 0.633 and a coefficient of determination of R^2^ = 0.980. The curve was fitted to the mean final inhibition values obtained from three technical replicate determinations at each concentration.

#### 3.1.2. TPC Screening

The TPC of the Lb aqueous extract was 10.84 ± 0.03 mg GAE/g dry extract (*n* = 3), as determined using the Folin–Ciocalteu assay.

#### 3.1.3. UHPLC–MS/MS Phytochemical Profile

Targeted UHPLC–MS/MS analysis revealed six quantifiable phenolic analytes or unresolved analyte groups in the Lb aqueous extract, whereas rutin was detected below the method’s limit of quantification. The most abundant signal corresponded to the unresolved 3,5-dihydroxybenzoic acid/3,4-dihydroxybenzoic acid group, with a concentration of 2.09 ± 0.60 µg/mL in the analyzed solution, corresponding to 0.209 ± 0.060 mg/g dry extract. This was followed by the p-coumaric acid/trans-p-coumaric acid group at 1.31 ± 0.04 µg/mL, corresponding to 0.131 ± 0.004 mg/g dry extract.

**Figure 3:**
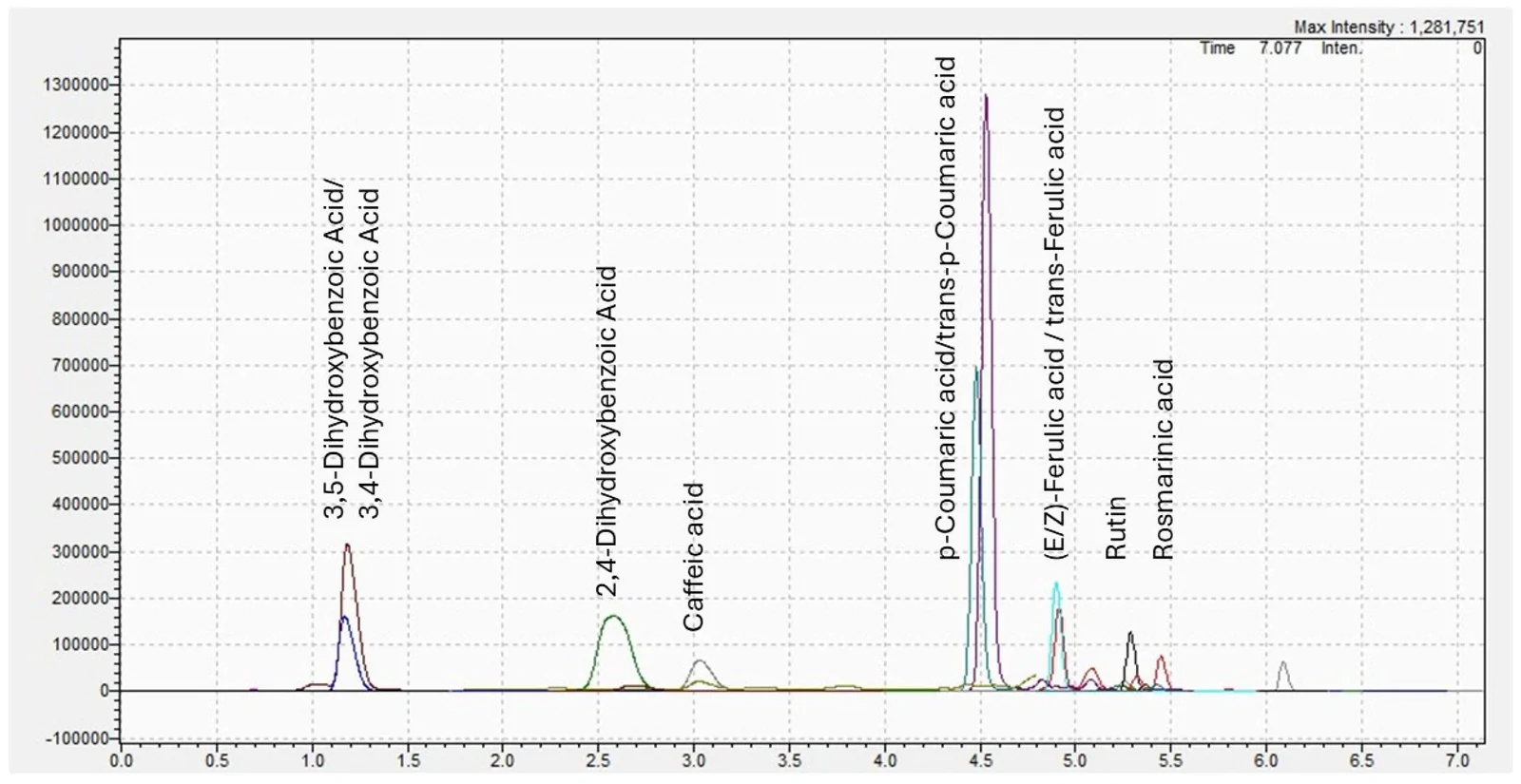
Representative UHPLC–MS/MS multiple-reaction-monitoring chromatogram of the phenolic compounds detected in the Lb aqueous extract.

Caffeic acid and the (E/Z)-ferulic acid/trans-ferulic acid group were quantified at 0.43 ± 0.04 and 0.36 ± 0.08 µg/mL, corresponding to 0.043 ± 0.004 and 0.036 ± 0.008 mg/g dry extract, respectively. Rosmarinic acid was detected at 0.19 ± 0.01 µg/mL (0.019 ± 0.001 mg/g dry extract), whereas 2,4-dihydroxybenzoic acid was present at 0.16 ± 0.04 µg/mL (0.016 ± 0.004 mg/g dry extract). Rutin was detected below the limit of quantification (<0.05 µg/mL; <0.005 mg/g dry extract). The reported values represent the mean ± SD of three technical determinations performed on the same analytical solution derived from the single extract batch; therefore, the observed variability reflects analytical repeatability rather than batch-to-batch variability. The corresponding chromatographic profile is presented in Figure 3, and the quantitative results are summarized in Table 1.

### 3.2. Biological In Vitro Screening

#### 3.2.1. Assessment of Acute Metabolic Viability Using 3D EpiAirway^TM^ Tissues

The three-dimensional EpiAirway^TM^ model consists of differentiated human tracheal and bronchial epithelial cells organized to reproduce selected structural and functional characteristics of the tracheobronchial airway epithelium. In the present study, tissue metabolic viability was evaluated under a single high-exposure condition. EpiAirway^TM^ tissues were exposed to 1000 µg/mL Lb aqueous extract for 24 h, and metabolic viability was subsequently assessed using the MTT assay.

**Figure 4:**
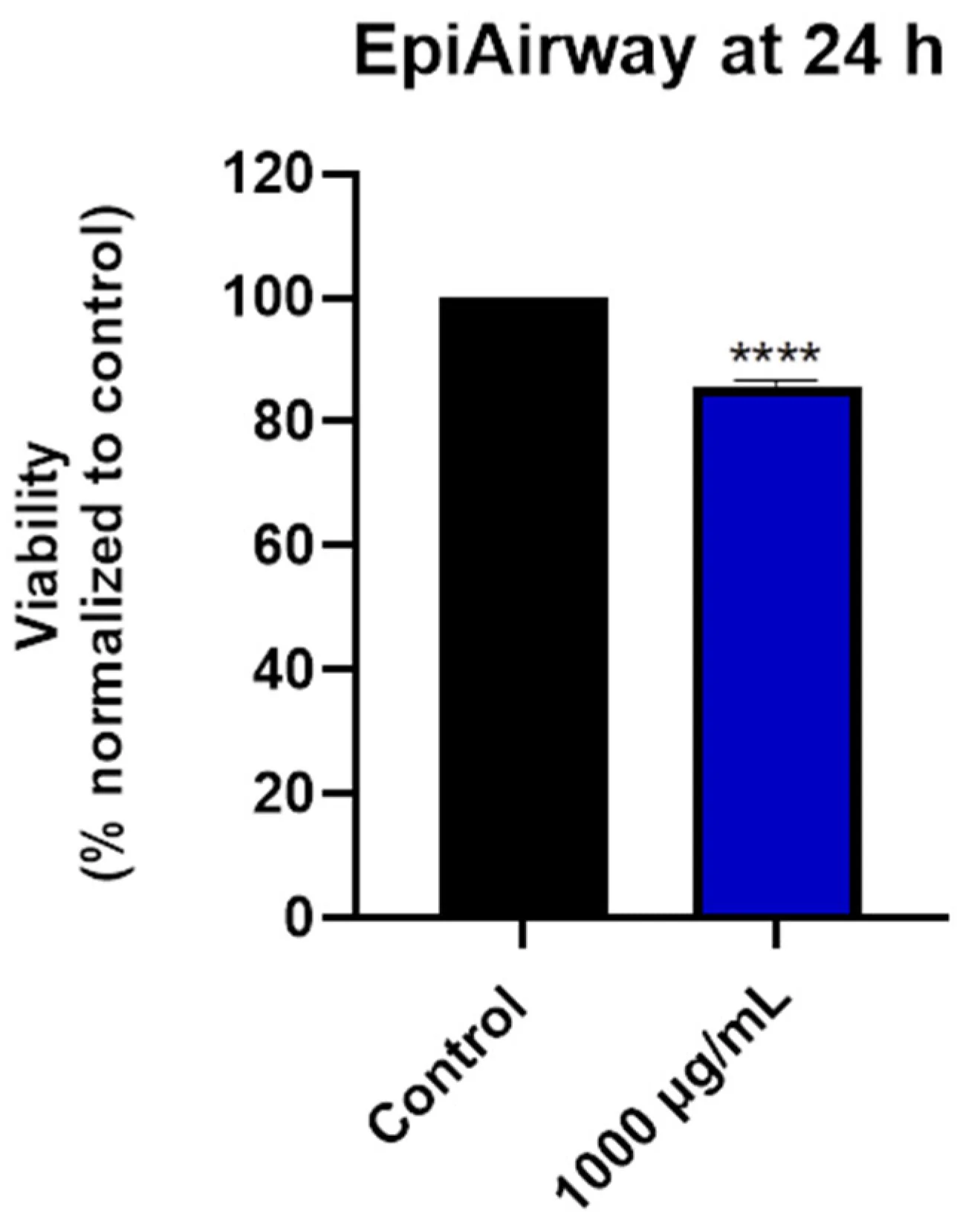
Assessment of the metabolic viability of reconstructed human respiratory epithelial tissue (EpiAirway^TM^) after 24 h of exposure to Lb aqueous extract at a concentration of 1000 µg/mL, as determined using the MTT assay. Results are expressed as percentages relative to the vehicle-treated control group, which was set to 100% viability. The experiment was performed in three independent biological experiments, each including three technical replicates. Technical replicates were averaged before statistical analysis, and data are presented as mean ± standard deviation (SD) of the three independent biological experiments. Statistical differences between the vehicle-treated control and the 1000 µg/mL extract-treated group were analyzed using an unpaired two-tailed *t*-test; **** *p* < 0.0001 compared with the control group.

Following exposure, EpiAirway^TM^ tissue metabolic viability was 85.87% relative to the control group. The reduction was statistically significant compared with the control (*p* < 0.0001) (Figure 4).

These data are limited to an acute MTT-based metabolic viability measurement conducted at a single extract concentration and a single exposure time. They should not be interpreted as evidence of respiratory safety, biocompatibility, or absence of other tissue-level effects. Additional concentrations, repeated or prolonged exposure, and complementary endpoints addressing tissue morphology, barrier integrity, and inflammatory responses would be required for a more comprehensive evaluation.

#### 3.2.2. Cell Viability

**Figure 5:**
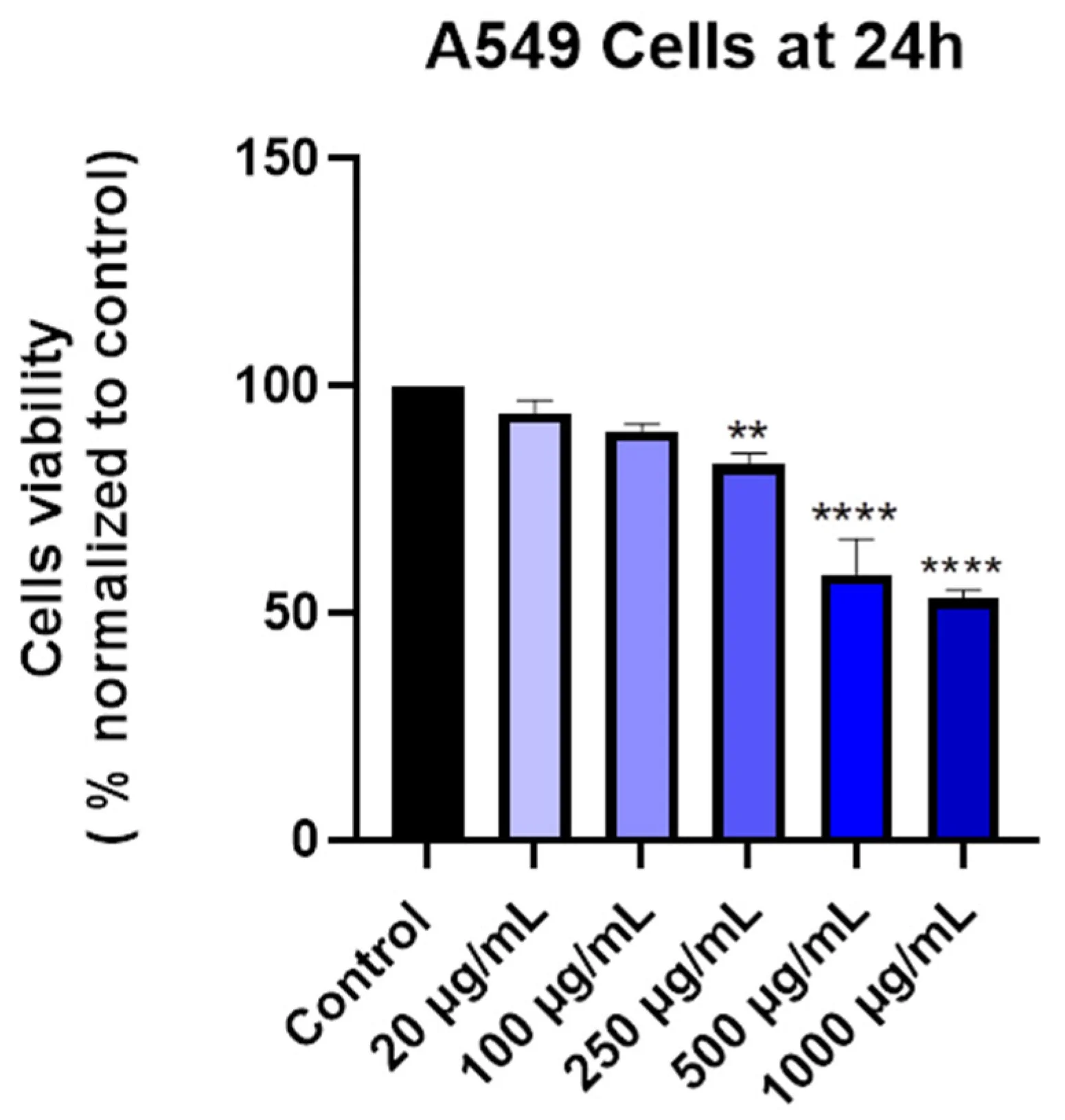
Effects of Lb aqueous extract on the viability of the human lung adenocarcinoma cell line (A549), after 24 h of treatment, as determined by the MTT assay. Results are expressed as percentages relative to the untreated control group (considered 100% viability). Data are presented as mean ± standard deviation (SD). Statistical analysis was performed using one-way ANOVA followed by Dunnett’s multiple comparisons test. Statistical significance relative to the control group is indicated as follows: ** *p* < 0.01 and **** *p* < 0.0001.

Following the EpiAirway^TM^ metabolic viability assessment, the effect of the Lb extract on A549 lung adenocarcinoma cells was evaluated using the MTT assay after 24 h of exposure to concentrations ranging from 20 to 1000 µg/mL. As shown in Figure 5, metabolic viability decreased in a concentration-dependent manner.

At the lowest concentrations, 20 and 100 μg/mL, cell viability remained relatively close to that of the control group (approximately 94% and 90%). At 250 µg/mL, metabolic viability decreased to approximately 83% and was significantly lower than that of the control group (*p* < 0.01). At 500 and 1000 µg/mL, metabolic viability decreased to approximately 59% and 53%, respectively, with both reductions reaching statistical significance (*p* < 0.0001). Marked cytotoxic effects were observed at the highest concentrations, with cell viability reduced to approximately 59% at 500 μg/mL and 53% at 1000 μg/mL. Both reductions were highly statistically significant (*p* < 0.0001) and fell below the viability threshold of 70%. The largest decline in metabolic viability occurred between 250 and 500 μg/mL, whereas the relatively small difference between 500 and 1000 μg/mL suggests a tendency toward a plateau within the tested concentration range.

#### 3.2.3. Microscopic Assessment of Cell Morphology

**Figure 6:**
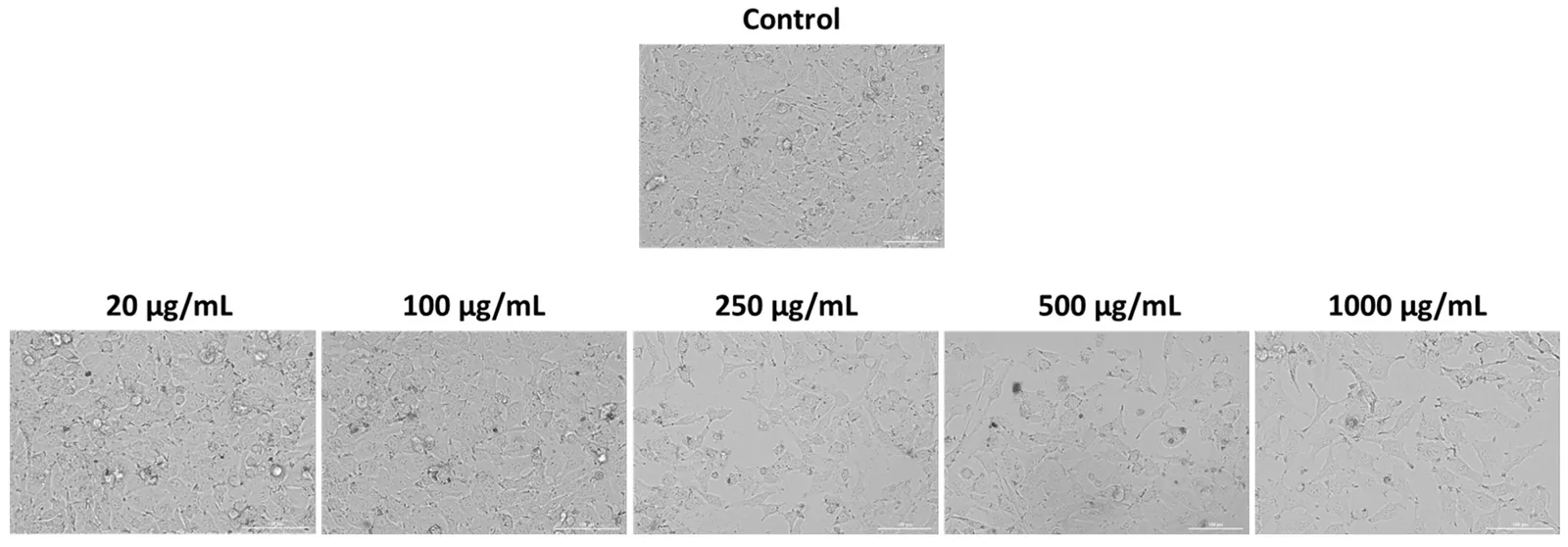
The morphological appearance of the A549 cell line following 24 h of treatment with Lb aqueous extract at concentrations ranging from 20 to 1000 µg/mL. The scale bar indicates 100 µm.

To further characterize the cytotoxic response, A549 cell morphology was evaluated by brightfield microscopy. Treatment induced concentration-dependent morphological alterations consistent with the cytotoxic profile obtained using the MTT assay (Figure 6). The control group was characterized by a confluent monolayer of cells exhibiting typical epithelial morphology, polygonal shape, and uniform density, with cells firmly adherent to the culture support.

Starting from a concentration of 100 µg/mL, minor alterations were observed, such as the progressive diminution of cellular confluence, which became increasingly pronounced with higher concentrations. Concurrently, the treated cells exhibited a loss of adhesion to the substrate, accompanied by characteristic morphological modifications, including the loss of the polygonal epithelial phenotype, cytoplasmic retraction, and cellular elongation, resulting in a fusiform morphology. The elevated concentrations tested (500 and 1000 µg/mL) additionally induced an expansion of intercellular spaces and the emergence of rounded, refractile (shiny) cells detaching from the culture substrate, alongside the presence of cellular debris. Furthermore, the concentration of 1000 µg/mL elicited the formation of membrane blebbing.

The observed morphological changes included reduced cellular confluence, loss of adhesion, cytoplasmic retraction, cellular elongation, increased intercellular spaces, cell rounding, detachment, membrane blebbing, and the presence of cellular debris. These alterations became progressively more pronounced at 500 and 1000 µg/mL.

#### 3.2.4. Neutral Red Uptake

To confirm the cytotoxic effect of Lb extract via a supplementary cellular endpoint, the viability of A549 cells was additionally evaluated utilizing the neutral red uptake (NRU) assay after a 24 h treatment period. In contrast to the MTT assay, which measures mitochondrial metabolic activity, the NRU assay assesses lysosomal membrane integrity and the cell’s capacity to retain the dye within functional lysosomes. The assay was therefore used as a complementary endpoint to the MTT measurement.

**Figure 7:**
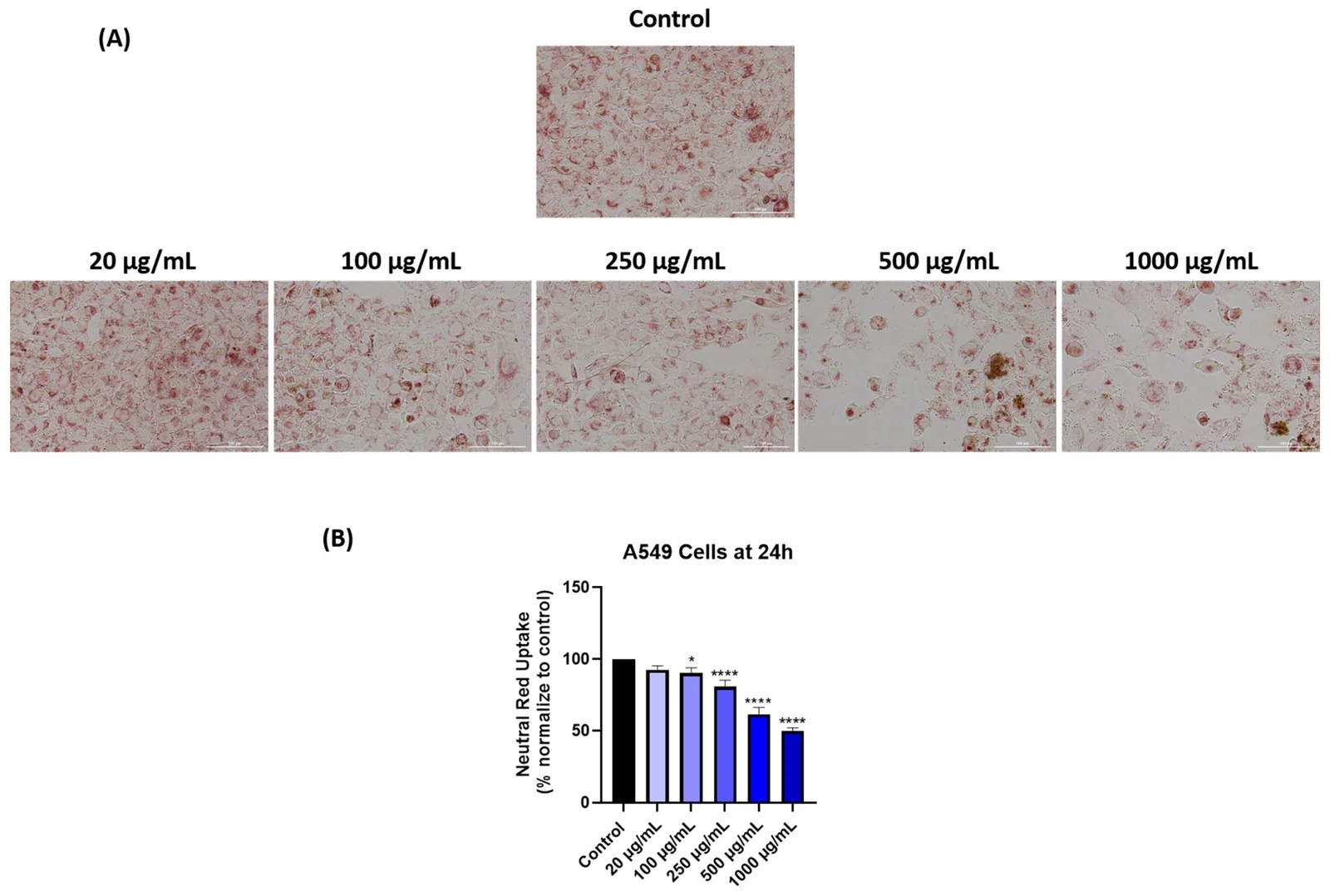
Assessment of the cytotoxic effect of Lb aqueous extract on A549 cells after 24 h of treatment, using the neutral red uptake (NRU) assay. (**A**) Representative microscopy images of cells stained with neutral red at concentrations ranging from 20 to 1000 µg/mL, compared to the untreated control group. (**B**) Quantification of cell viability, expressed as a percentage relative to the untreated control group (100%). Data are presented as mean ± standard deviation (SD). Statistical differences compared to the control were analyzed using one-way ANOVA, followed by Dunnett’s post hoc test. * *p* < 0.05 and **** *p* < 0.0001 compared to the control group.

Microscopic examination of the cells, as presented in Figure 7A, revealed a reduction in neutral red uptake beginning at a concentration of 100 µg/mL. This phenomenon was evidenced by a decrease in staining intensity and the number of stained cells, effects that became more pronounced with increasing tested concentrations. These qualitative observations were corroborated by quantitative analysis (Figure 7B), which demonstrated that the decrease in cell viability is concentration-dependent, with viability values declining from approximately 93% at 20 µg/mL to approximately 50% at 1000 µg/mL. The reduction was statistically significant starting at 100 µg/mL (*p* < 0.05), and attained a highly significant level at concentrations of 250, 500, and 1000 µg/mL (*p* < 0.0001) in comparison to the control group.

The quantitative NRU findings paralleled the concentration-dependent reduction observed in the MTT assay, although the two assays evaluated different cellular endpoints.

#### 3.2.5. Mitochondrial Membrane Potential (ΔΨm) Assay—JC-1 Staining

To assess the influence of Lb extract on mitochondrial membrane potential in A549 cells, the JC-1 assay was utilized. This lipophilic cationic dye selectively accumulates within functional mitochondria exhibiting an intact membrane potential, forming aggregates that emit red fluorescence (J-aggregates). Conversely, when the membrane potential is compromised through mitochondrial depolarization, the dye remains in the cytoplasm in its monomeric form, which emits green fluorescence. Therefore, the ratio of fluorescence intensity between the aggregates and the monomers (red/green) provides a direct measure of mitochondrial integrity.

**Figure 8:**
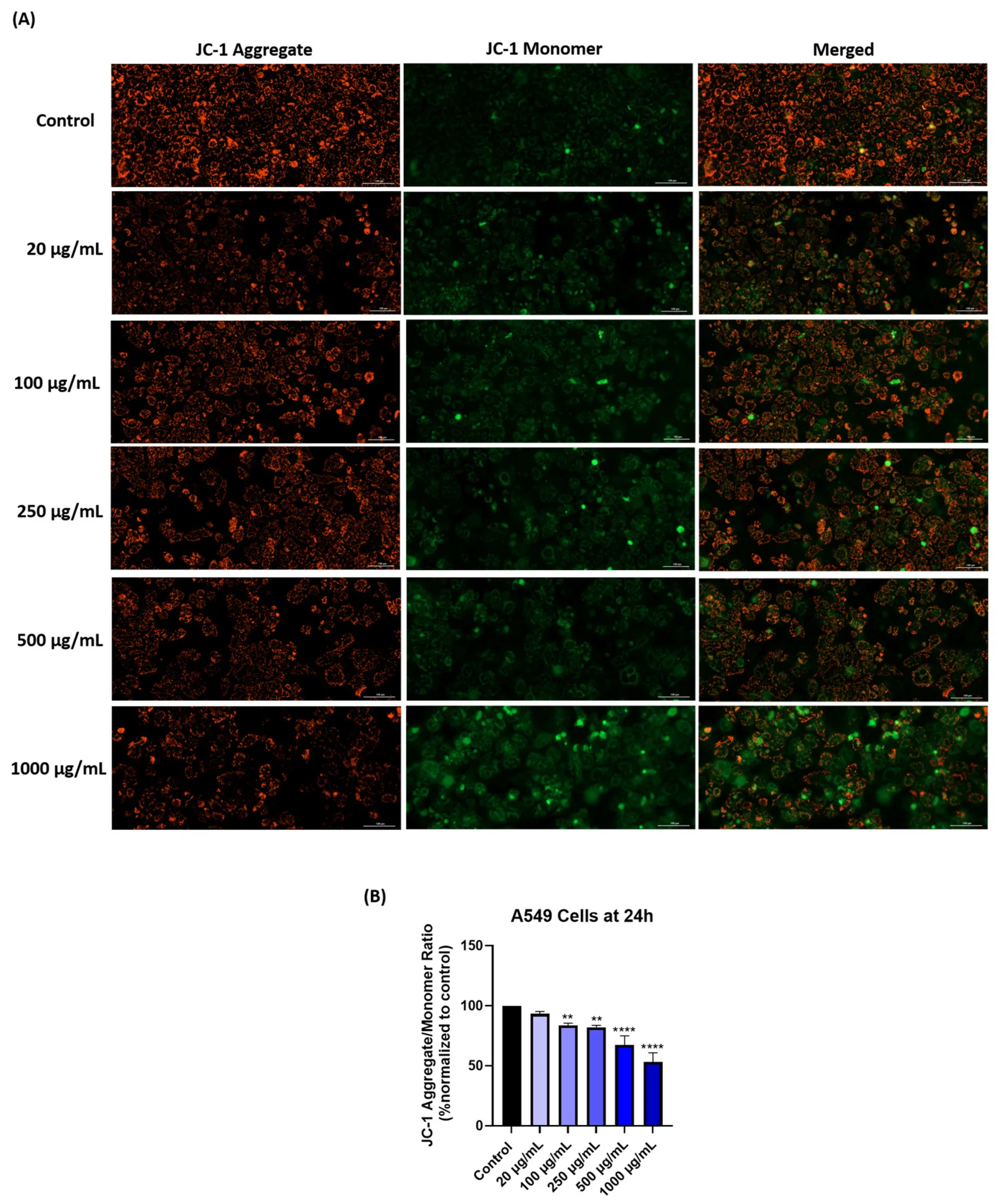
Assessment of the mitochondrial membrane potential of A549 cells after 24 h of treatment with Lb aqueous extract, using the JC-1 assay. (**A**) Representative fluorescence microscopy images illustrating JC-1 aggregates (red fluorescence, polarized mitochondria), JC-1 monomers (green fluorescence, depolarized mitochondria), and merged images, at concentrations ranging from 20 to 1000 µg/mL, compared to the untreated control group. (**B**) Quantification of the JC-1 aggregate-to-monomer fluorescence ratio, calculated directly from the Cytation 5 microplate reader measurements as red fluorescence at 590 nm divided by green fluorescence at 529 nm. The resulting ratios were normalized to the mean ratio of the untreated control group, which was set to 100%. Data are presented as mean ± standard deviation (SD). Statistical differences compared to the control were analyzed using one-way ANOVA followed by Dunnett’s post hoc test. ** *p* < 0.01 and **** *p* < 0.0001.

Microscopic analysis revealed a proportional increase in green fluorescence with higher tested concentrations, alongside a reduction in red fluorescence. This pattern indicates concentration-dependent mitochondrial depolarization, becoming more noticeable as the concentration rises, especially at 1000 μg/mL. The merged images demonstrate a concentration-dependent shift from predominantly red fluorescence in controls to more green fluorescence at higher doses. At 500 and 1000 μg/mL, there is also a decrease in cell density, with a change in aggregate signal distribution from dense and reticular to punctate or fragmented forms. Rounded cells displaying intense green fluorescence were observed at the highest concentrations, together with reduced cell density and altered distribution of the red aggregate signal (Figure 8A).

Quantification of the JC-1 aggregate-to-monomer ratio demonstrated a concentration-dependent decrease from approximately 94% at 20 µg/mL to approximately 53% at 1000 µg/mL. The reduction was statistically significant at 100 and 250 µg/mL (*p* < 0.01) and at 500 and 1000 µg/mL (*p* < 0.0001). These results demonstrate a concentration-dependent reduction in the JC-1 aggregate-to-monomer fluorescence ratio following treatment with the Lb extract (Figure 8B).

#### 3.2.6. Analysis of Nuclear and Mitochondrial Aspects Using Fluorescence Staining

**Figure 9:**
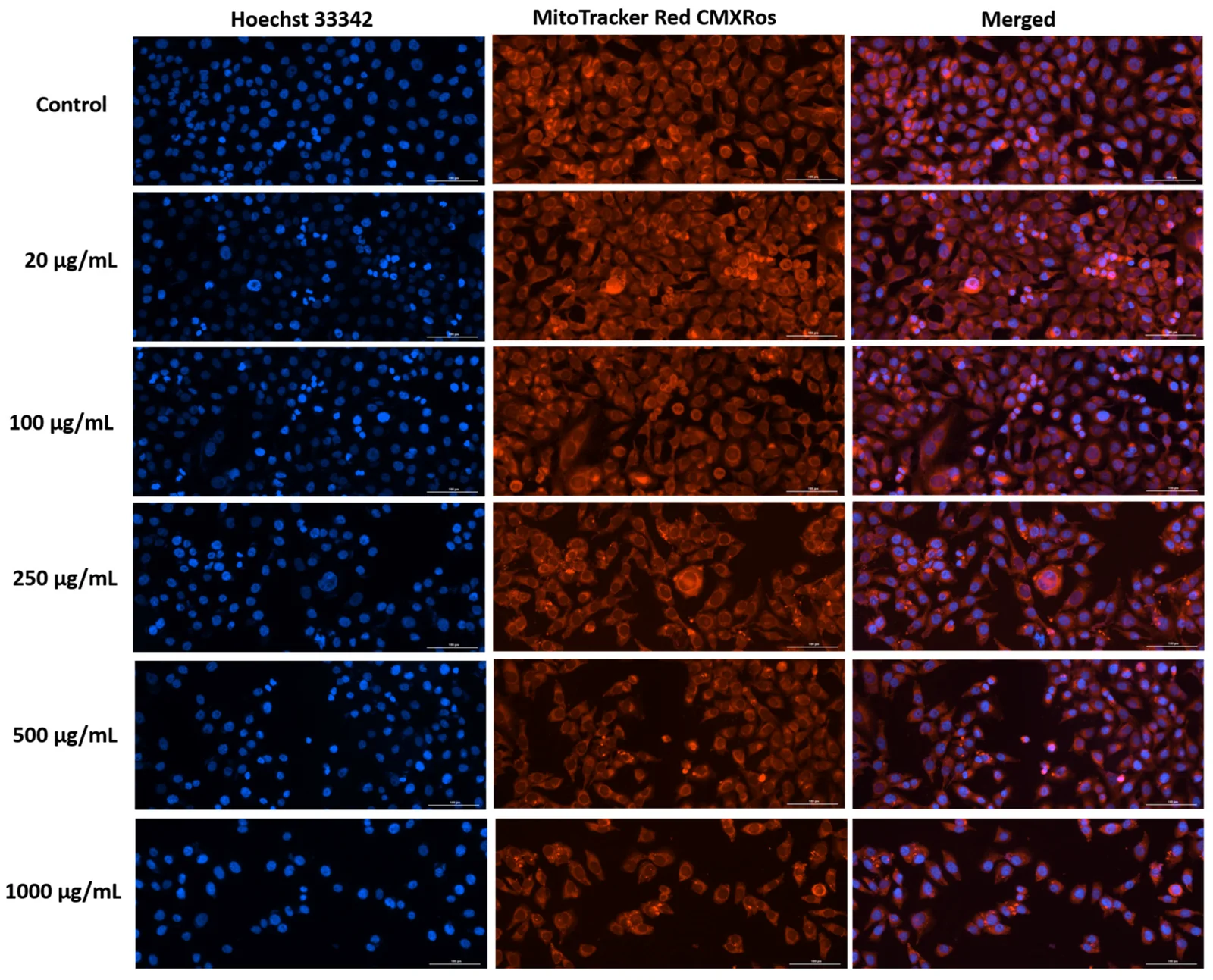
Assessment of the effects of Lb aqueous extract on mitochondrial and nuclear morphology in A549 cells after 24 h of treatment. Representative fluorescence microscopy images of A549 cells exposed to Lb extract at concentrations ranging from 20 to 1000 μg/mL. Images were obtained at 20× magnification, and the scale bar was 100 µm.

For a more detailed characterization of the effects induced by Lb extract at the subcellular level, A549 cells were subjected to double fluorescent staining, using Hoechst 33342 to accentuate nuclear morphology and MitoTracker Red CMXRos to visualize the mitochondrial network. The observed effects suggested nuclear and mitochondrial alterations proportional to the dose, aligning with previously demonstrated cytotoxic effects and mitochondrial dysfunction (Figure 9).

At the nuclear level, control cells exhibited nuclei with typical morphology, oval-shaped, with chromatin evenly distributed and uniformly stained. Following Lb extract application, nuclear changes dependent on concentration were observed. Chromatin condensation appeared, evidenced by increased intensity and non-homogeneous blue fluorescence, along with the appearance of rounded nuclei starting at a concentration of 20 µg/mL. Additionally, a reduction in nuclear density was noted, manifested by a decrease in the number of nuclei as the concentration increased, as well as a tendency for nuclei to shrink through condensation or pyknosis at higher concentrations. Following treatment, concentration-dependent changes were observed, including increased and heterogeneous Hoechst fluorescence, chromatin condensation, rounded and smaller nuclei, and reduced nuclear density. These alterations were more pronounced at the highest tested concentrations.

At the mitochondrial level, a series of changes were observed, such as the intensity and distribution of the fluorescent signal, which depended on the concentration. Compared to control cells characterized by a reticular, filamentous appearance of the mitochondrial network, treated cells showed an increase in mitochondrial fluorescence intensity, along with a change in mitochondrial morphology toward a rounded appearance and a reduction in their size. These alterations were notably more pronounced at concentrations of 500 and 1000 μg/mL. However, these qualitative observations do not establish a mitochondrial-dependent apoptotic mechanism, which would require confirmation using apoptosis-specific molecular or flow-cytometric endpoints.

#### 3.2.7. Acridine Orange/Propidium Iodide (AO/PI) Assay

**Figure 10:**
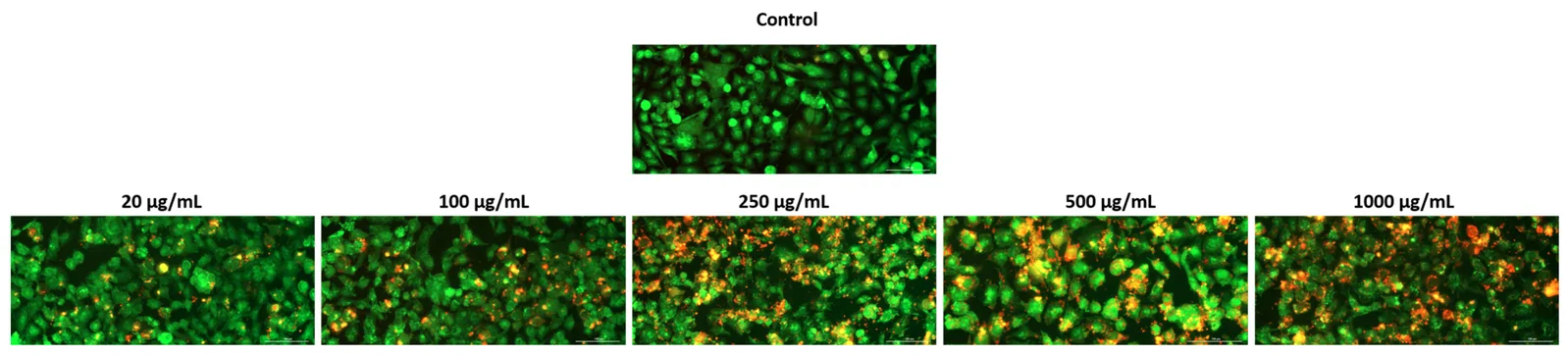
Representative fluorescence microscopy images of A549 cells treated with Lb aqueous extract at concentrations ranging from 20 to 1000 μg/mL for 24 h and stained with acridine orange/propidium iodide (AO/PI). Images were acquired at 20× magnification. The scale bar indicates 100 μm.

To assess plasma membrane integrity and AO/PI staining patterns, A549 cells were examined after 24 h of treatment. Acridine orange stained the cell population green, whereas propidium iodide produced orange-red fluorescence in cells with compromised plasma membranes (Figure 10).

Microscopic examination demonstrated a pattern of concentration-dependent alterations. The control group primarily exhibited a green cell population, characterized by uniform fluorescence and typical morphology indicative of viable cells, without evidence of plasma membrane damage. Treatment with the Lb extract resulted in a concentration-dependent increase in orange-red fluorescence and a corresponding reduction in the green-stained cell population. The most pronounced changes were observed at 500 and 1000 µg/mL. Condensed or fragmented nuclear structures were also observed in some cells displaying orange-red fluorescence.

These observations demonstrate that the reduction in cellular viability was accompanied by a concentration-dependent increase in cells with compromised plasma membranes. Because the AO/PI assessment was qualitative, the relative contributions of apoptosis and necrosis could not be quantified or definitively distinguished.

#### 3.2.8. Colony Formation Assay

The clonogenic capacity of A549 cells was assessed to determine the influence of Lb extract on their long-term proliferative potential following a 24 h treatment period, followed by a 10-day growth phase. Prior evaluations, such as short-term viability assays, including MTT and NRU, represent the metabolic status at 24 h. Conversely, the colony formation assay measures the sustained reproductive capacity of the cells, specifically, the ability of a single cell to survive and form a colony. This assay was used to quantify the ability of treated cells to retain colony-forming capacity after removal of the extract.

**Figure 11:**
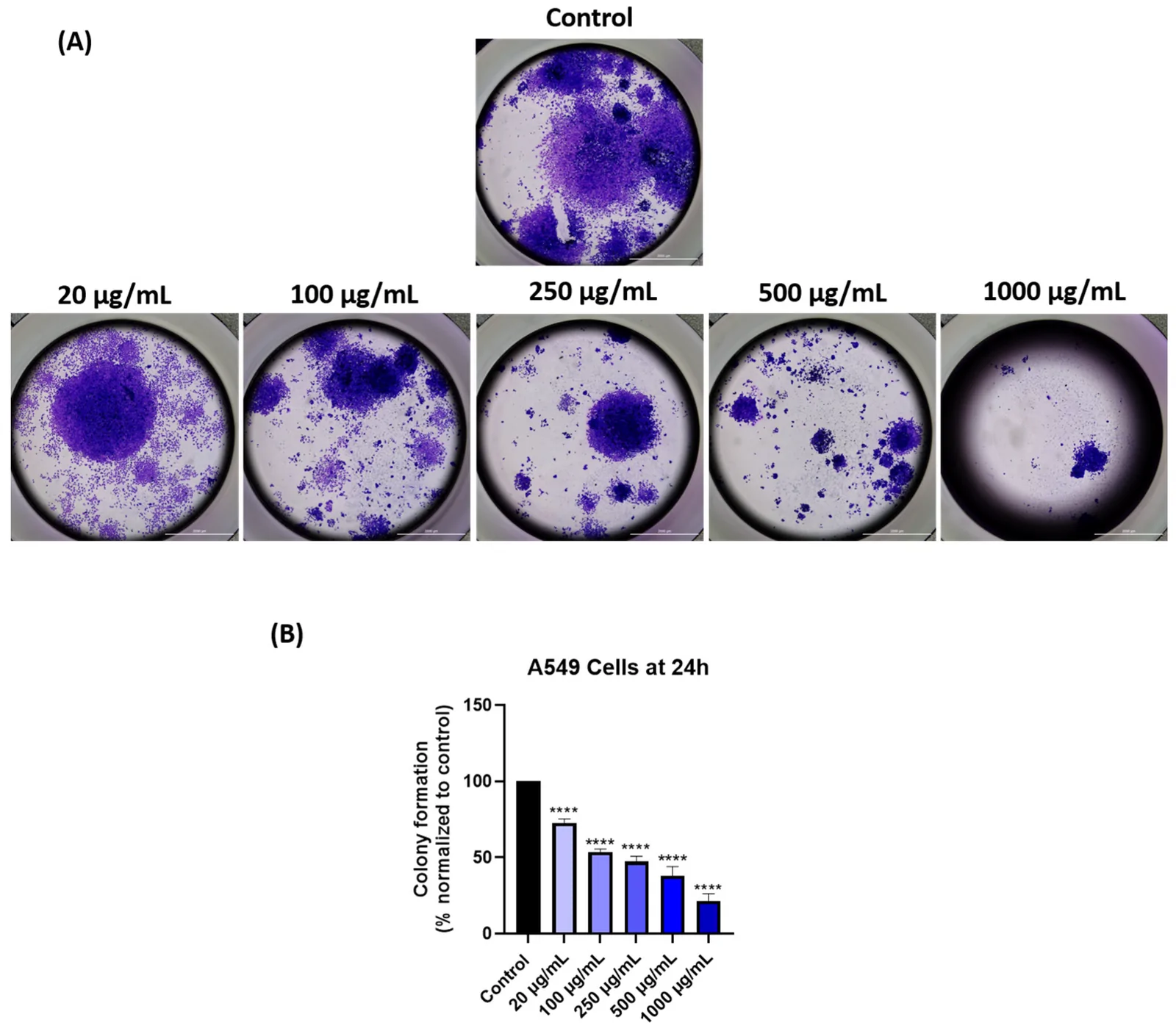
Effect of Lb aqueous extract on the clonogenic potential of A549 cells after 24 h of treatment followed by a 10-day colony-growth period. (**A**) Representative images of colony formation; the scale bar represents 2000 µm. (**B**) Quantification of colony formation after 24 h exposure to Lb extract, expressed as a percentage relative to the untreated control group (100%). Data are presented as mean ± standard deviation (SD). Statistical differences compared with the control were analyzed using one-way ANOVA followed by Dunnett’s post hoc test; **** *p* < 0.0001 compared with the control group.

In Figure 11A, it is observed that Lb extract induces a concentration-dependent reduction in colony-forming ability compared to the untreated control group. This effect is manifested by a decrease in the number and size of colonies, proportional to the tested concentration. Additionally, through quantitative analysis (Figure 11B), it was shown that compared to the control, the clonogenic capacity is reduced to approximately 73% at a concentration of 20 μg/mL, and to 21% at a concentration of 1000 μg/mL.

At 20 µg/mL, metabolic viability measured after 24 h was approximately 94%, whereas clonogenic capacity measured after the subsequent 10-day growth period was approximately 73%. At 1000 µg/mL, clonogenic capacity decreased to approximately 21% of the untreated control value. Overall, transient exposure to the Lb extract resulted in a concentration-dependent reduction in the subsequent colony-forming capacity of A549 cells.

## 4. Discussion

The present study integrated phytochemical and antioxidant characterization with biological evaluation in a reconstructed human tracheobronchial tissue model and A549 lung adenocarcinoma cells. Its principal contribution lies in the assessment of the same aqueous *Melissa officinalis* leaf extract using complementary analytical, acute metabolic, lysosomal, mitochondrial, morphological, membrane-integrity, and clonogenic endpoints. This integrated approach provides a broader characterization than an isolated antioxidant or short-term viability assay, while the findings remain exploratory.

The antioxidant activity and phenolic composition of *Melissa officinalis* preparations are strongly influenced by plant origin and plant part, solvent polarity, solid-to-liquid ratio, extraction temperature and duration, energy input, post-extraction processing, and the basis used for result normalization [16,18,32,33,34,35,36,37,38,39]. The extraction procedure employed in the present study combined 48 h of orbital extraction at 25 °C with 60 min of probe sonication. Although these conditions may enhance the release of intracellular and cell-wall-associated constituents, prolonged extraction and the localized energy generated during probe sonication may also promote oxidation, hydrolysis, or degradation of susceptible phytochemicals. Consequently, the phytochemical profile obtained in the present study may differ from those produced by infusion, decoction, shorter conventional procedures, or microwave-assisted extraction. However, these methods are not directly equivalent because their time–temperature profiles, energy input, plant-to-solvent ratios, concentration procedures, and drying conditions may differently influence compound recovery and stability.

In the present study, the Lb aqueous extract exhibited concentration-dependent DPPH radical-scavenging activity, increasing from 37.56% at 0.5 mg/mL to 80.01% at 10 mg/mL. Nonlinear regression yielded an estimated IC_50_ of 1.37 mg/mL (95% CI: 1.00–1.88 mg/mL), with a Hill slope of 0.633, indicating a gradual concentration–response relationship. The total phenolic content was 10.84 ± 0.03 mg GAE/g dry extract. Considerable variability has been reported among aqueous or water-based *M. officinalis* preparations, with DPPH-derived IC_50_/EC_50_ values ranging from approximately 4.76 µg/mL to 4.31 mg/mL and reported TPC values ranging from approximately 5.6 to more than 300 mg GAE/g, depending on the extraction procedure and the normalization basis used [18,37,38,39,40,41,42,43,44]. These ranges are descriptive rather than directly comparable, because plant material, extraction and drying conditions, DPPH assay parameters, curve-fitting methods, and reporting units can substantially influence the resulting values. The antioxidant response observed in the present study is therefore likely attributable to the combined contribution of multiple hydrophilic constituents rather than to a single predominant compound.

Targeted UHPLC–MS/MS analysis provided a partial phenolic profile of the Lb aqueous extract. The highest quantified signal corresponded to the unresolved 3,5-dihydroxybenzoic acid/3,4-dihydroxybenzoic acid group (2.09 ± 0.60 µg/mL), followed by the p-coumaric acid/trans-p-coumaric acid group (1.31 ± 0.04 µg/mL). Caffeic acid, the (E/Z)-ferulic acid/trans-ferulic acid group, rosmarinic acid, and 2,4-dihydroxybenzoic acid were detected at lower concentrations, whereas rutin was detected below the limit of quantification. Gallic acid, chlorogenic acid, resveratrol, quercetin, and genistein were not detected under the applied conditions, although their presence below the method’s limit of detection cannot be excluded. Because the method targeted a predefined analyte panel, these findings represent only the monitored phenolic fraction rather than the complete phytochemical composition of the extract. In addition, incomplete chromatographic resolution prevented reliable discrimination between the 3,4-/3,5-dihydroxybenzoic acid, p-coumaric acid/trans-p-coumaric acid, and (E/Z)-ferulic acid/trans-ferulic acid isomers; therefore, the corresponding signals were assigned and quantified collectively based on their characteristic MRM transitions and retention behavior relative to authentic standards.

The sum of the six quantified analytes or unresolved analyte groups was approximately 0.454 mg/g dry extract, which was lower than the total phenolic content of 10.84 mg GAE/g dry extract. However, these values are not directly comparable because the Folin–Ciocalteu assay estimates overall reducing capacity and may respond to both phenolic and non-phenolic reducing constituents, whereas UHPLC–MS/MS quantifies only the predefined analytes. This difference therefore reflects the distinct selectivity and reporting principles of the two methods and suggests the presence of additional unmonitored phenolic constituents. The detected phenolic acids were broadly consistent with previously reported *M. officinalis* profiles, although their relative abundance varies considerably [18,38,39,45,46]. Such variability may reflect differences in plant provenance, genotype, harvest stage, drying and extraction conditions, analytical platforms, and result normalization. Because the individual contribution of each compound was not assessed, the observed antioxidant and cellular effects should be attributed to the whole extract, potentially involving additive or interactive effects among detected and unmonitored constituents. Compound-specific activity would require evaluation using isolated standards, standardized mixtures, or activity-guided fractionation.

The second objective was to characterize the in vitro biological response to the Lb aqueous extract through (i) acute metabolic viability assessment in the EpiAirway^TM^ three-dimensional tracheobronchial tissue model under a single exposure condition and (ii) evaluation of cytotoxic and sustained antiproliferative effects in A549 human lung adenocarcinoma cells. The concentration range of 20–1000 μg/mL was selected based on previous studies investigating Lb extracts in tumor cell models [17,18,21,47].

Following a single 24 h exposure to 1000 µg/mL Lb aqueous extract, EpiAirway^TM^ tissue metabolic viability remained at 85.87% relative to the vehicle-treated control, indicating no marked acute metabolic cytotoxicity under the specific conditions tested. EpiAirway^TM^ reproduces selected characteristics of differentiated human tracheobronchial epithelium [25], and a previous study similarly reported high viability in HaCaT keratinocytes exposed for 24 h to an aqueous Lb leaf extract at concentrations up to 1000 µg/mL [18]. However, this finding should not be interpreted as evidence of comprehensive respiratory safety or tumor selectivity, because EpiAirway^TM^ and A549 differ in tissue origin, dimensionality, cellular composition, culture medium, and exposure configuration. Accordingly, demonstration of preferential tumor cytotoxicity would require concentration-matched comparisons between malignant and appropriate non-malignant human alveolar epithelial models under equivalent experimental conditions.

In A549 lung adenocarcinoma cells, the Lb aqueous extract produced a concentration-dependent reduction in metabolic viability after 24 h, reaching approximately 53% at 1000 µg/mL. A549 cells are widely used as a two-dimensional model of non-small-cell lung adenocarcinoma [48], and comparable concentration-dependent effects of *M. officinalis* extracts have been reported in other tumor cell models [17,47,49,50,51]. Although A549 cells harbor the KRAS G12S mutation and are reported to be wild-type for EGFR and TP53 [52,53], genotype-specific sensitivity and downstream apoptotic signaling were not investigated; therefore, the observed response cannot be attributed to KRAS status or a TP53-dependent mechanism [54,55].

The reduction in A549 metabolic viability was accompanied by concentration-dependent morphological alterations, particularly at the higher extract concentrations, including loss of cell-to-cell adhesion, cell shrinkage, rounding, and membrane blebbing [56]. Comparable apoptotic-like morphological changes have been reported following exposure of A375 melanoma, PC-3 and LNCaP prostate adenocarcinoma, and MCF-7 breast adenocarcinoma cells to different Lb extracts [18,57]. Consistently, the NRU assay demonstrated a concentration-dependent reduction in dye uptake, indicating impaired lysosomal integrity and cellular viability [58,59,60]. Nevertheless, neither cellular morphology nor reduced neutral red retention is sufficient to identify a specific mode of cell death.

Because lysosomal dysfunction may contribute to mitochondrial injury [59], mitochondrial membrane potential was assessed as an additional mechanistic endpoint [61]. Lb extract treatment produced a concentration-dependent decrease in the JC-1 aggregate-to-monomer ratio, which reached approximately 53% of the control value at 1000 μg/mL, supporting mitochondrial membrane depolarization. Similar mitochondrial effects have been reported in other cancer cell models treated with Lb extract [62]. However, mitochondrial depolarization is not specific to apoptosis and does not independently confirm activation of the intrinsic apoptotic pathway. Moreover, because the JC-1 ratio was not normalized to cell number or viability, the reduced cell density observed at the highest concentrations may have influenced the magnitude of the recorded response.

Hoechst 33342 and MitoTracker staining provided complementary qualitative information on nuclear and mitochondrial alterations following 24 h of Lb extract exposure. Treatment induced concentration-dependent chromatin condensation, nuclear shrinkage, rounded nuclei, and changes in mitochondrial morphology, consistent with the mitochondrial dysfunction observed in the JC-1 assay [63,64,65,66]. However, these qualitative alterations are not specific enough to confirm apoptosis. Similar apoptotic-like nuclear changes were reported in A375 melanoma cells treated with Lb ethanolic extract [47].

AO/PI staining revealed a concentration-dependent increase in cells with compromised plasma membranes and staining patterns compatible with apoptotic-like or necrotic injury [67,68]. However, because this assessment was qualitative and apoptosis-specific molecular endpoints were not evaluated, the relative contributions of apoptosis and necrosis could not be established. Similar propidium iodide staining patterns have been reported in other cancer cell lines treated with a hydroalcoholic *M. officinalis* extract [49].

A particularly relevant contribution of the present study is the inclusion of the colony formation assay, which assessed the ability of A549 cells to retain reproductive capacity after removal of the extract [69,70]. A transient 24 h exposure produced a concentration-dependent reduction in subsequent colony formation during the 10-day growth period. The reduction was evident even at concentrations associated with relatively limited changes in short-term metabolic viability, indicating that an acute MTT measurement alone did not fully capture the later effect on reproductive survival. Similar persistent inhibition of colony formation has been reported in HCT116 cells treated with *M. officinalis* extract [71]. These findings demonstrate that the biological response extended beyond the initial exposure period, although they do not by themselves establish therapeutic antitumor efficacy.

Overall, the study shows that the investigated aqueous *M. officinalis* leaf extract combines measurable antioxidant activity with concentration-dependent short-term cytotoxic and sustained antiproliferative effects in A549 cells. The integration of complementary endpoints strengthens the evidence that the response involved multiple cellular compartments rather than an isolated alteration in one assay. At the same time, the single extract batch, the absence of matched non-malignant alveolar cells, the limited EpiAirway^TM^ design, and the lack of apoptosis-specific molecular quantification restrict mechanistic and translational conclusions. The findings should therefore be regarded as a basis for further standardized and mechanistically focused investigation rather than as evidence of an established anticancer agent, therapeutic adjuvant, or respiratory-safe preparation.

### Study Limitations and Future Perspectives

Several limitations of the present study should be addressed in subsequent research.

First, all phytochemical and biological analyses were conducted using a single independently prepared extract batch obtained from plant material collected at one location and one phenological stage. Consequently, the present study does not capture potential variability associated with plant provenance, genotype, year-to-year climatic conditions, soil characteristics, harvest timing, drying conditions, or extraction reproducibility. Although the cell-based assays were repeated in three independent biological experiments, all experiments used the same extract batch; therefore, they establish experimental repeatability for the investigated batch but not batch-to-batch reproducibility. Moreover, residual moisture, water activity, and time-dependent chemical or microbiological stability were not assessed. The results therefore characterize the extract at the time of analysis and do not establish storage stability or shelf life. Future studies should evaluate multiple independent plant collections and extract batches and compare extraction yield, targeted and untargeted phytochemical profiles, antioxidant activity, and biological responses to quantify inter-batch variability and establish standardization and batch-acceptance criteria.

Second, the biological evaluation was performed using a single malignant lung cell line, A549, and did not include additional non-small-cell lung cancer models with distinct molecular backgrounds. Moreover, no non-malignant lung epithelial cell line was evaluated under culture and exposure conditions directly comparable to those used for A549 cells. Although the EpiAirway^TM^ model provides a physiologically relevant reconstructed human respiratory epithelium, it differs from the A549 monolayer model in dimensionality, cellular composition, culture medium, and exposure configuration. Consequently, the differences observed between the two systems cannot establish tumor-selective cytotoxicity or define a therapeutic window. Accordingly, the EpiAirway^TM^ result should be interpreted as an independent assessment of acute tissue metabolic viability under one exposure condition and not as a normal-cell comparator for calculating or inferring a tumor-selectivity index.

Third, several methodological factors related to treatment solution preparation should be considered. The extract stock solution was prepared in ultrapure water and represented up to 10% (*v*/*v*) of the final treatment solution at the highest tested concentration, resulting in a corresponding dilution of the assay medium. The pH and osmolality of the final treatment solutions were not measured, and cell-free extract controls were not included to assess potential direct interference with the colorimetric and fluorescence-based endpoints. Consequently, contributions from medium dilution, altered physicochemical properties, extract absorbance or autofluorescence, or direct chemical interactions with assay reagents cannot be completely excluded, particularly at the highest concentrations. Future studies should incorporate appropriately matched vehicle controls, cell-free interference controls, and direct measurements of treatment solution pH and osmolality.

Fourth, EpiAirway^TM^ metabolic viability was assessed only at the highest tested concentration and after a single 24 h exposure period, using the MTT assay as the only tissue endpoint. Additional analyses of tissue morphology, barrier integrity, inflammatory responses, and repeated or prolonged exposure would provide a more comprehensive characterization of the respiratory tissue response. Similarly, the short-term A549 experiments were limited to 24 h, and because metabolic viability remained approximately 53% at 1000 µg/mL, an IC_50_ value could not be reliably determined within the investigated concentration range.

Fifth, mitochondrial depolarization, nuclear and cellular morphological alterations, and AO/PI staining patterns were compatible with apoptosis-like cellular injury; however, these functional and qualitative endpoints did not establish apoptosis as the predominant mode of cell death or confirm activation of a specific mitochondrial apoptotic pathway. Future studies should include quantitative flow-cytometric and molecular analyses, such as Annexin V/PI staining, caspase activation, cytochrome c release, and assessment of the BAX/BCL-2 balance.

## 5. Conclusions

The present study provides an integrated phytochemical, antioxidant, and biological characterization of an aqueous *Melissa officinalis* leaf extract. The extract exhibited concentration-dependent DPPH radical-scavenging activity, with an estimated IC_50_ of 1.37 mg/mL, and a total phenolic content of 10.84 ± 0.03 mg GAE/g dry extract. Targeted UHPLC–MS/MS analysis identified several hydroxybenzoic and hydroxycinnamic acid derivatives, with the unresolved 3,5-dihydroxybenzoic acid/3,4-dihydroxybenzoic acid group and the p-coumaric acid/trans-p-coumaric acid group representing the most abundant quantified analytes. Caffeic, ferulic, and rosmarinic acids were also quantified, whereas rutin was detected below the limit of quantification.

Following 24 h of exposure to 1000 µg/mL extract, EpiAirway^TM^ tissue metabolic viability remained at 85.87% under the single experimental condition tested. In A549 lung adenocarcinoma cells, the extract produced concentration-dependent reductions in metabolic viability and neutral red uptake, together with mitochondrial membrane depolarization and cellular, nuclear, and mitochondrial alterations. AO/PI staining demonstrated a concentration-dependent increase in cells with compromised plasma membranes, while transient exposure substantially reduced subsequent clonogenic capacity.

Taken together, these findings indicate that the aqueous *M. officinalis* leaf extract possesses measurable antioxidant activity and produces short-term cytotoxic and sustained antiproliferative effects in A549 cells. However, the EpiAirway^TM^ and A549 results represent responses obtained in two biologically and methodologically distinct experimental systems. Because no matched non-malignant human alveolar epithelial model was evaluated under equivalent culture and exposure conditions, the present study does not demonstrate preferential cytotoxicity toward tumor cells or define a therapeutic window. The cellular findings are compatible with mitochondrial involvement and apoptosis-like injury, but they do not establish a specific cell-death mechanism. Further studies using matched malignant and non-malignant lung epithelial models and quantitative molecular endpoints are required before tumor selectivity or a specific mechanism of cell death can be established.

## Figures and Tables

**Table 1:** Targeted phenolic compounds analyzed in the Lb aqueous extract by UHPLC–MS/MS.

Analyte or Analyte Group	Concentration in the Analyzed Solution [µg/mL]	Concentration Normalized to Dry Extract [mg/g Dry Extract]	MRM Transition (*m*/*z*)	Retention Time (min)
3,5-Dihydroxybenzoic Acid/ 3,4-Dihydroxybenzoic Acid	2.09 ± 0.60	0.209 ± 0.060	153.030 → 108.900	1.18
2,4-Dihydroxybenzoic Acid	0.16 ± 0.04	0.016 ± 0.004	153.030 → 108.900	2.72
Caffeic acid	0.43 ± 0.04	0.043 ± 0.004	179.040 → 135.050	3.03
p-Coumaric acid/ trans-p-Coumaric acid	1.31 ± 0.04	0.131 ± 0.004	163.050 → 119.050	4.53
(E/Z)-Ferulic acid/ trans-Ferulic acid	0.36 ± 0.08	0.036 ± 0.008	193.060 → 134.100	4.92
Rutin	<LOQ *	<0.005 *	609.150 → 300.100	5.29
Rosmarinic acid	0.19 ± 0.01	0.019 ± 0.001	359.080 → 161.100	5.49
Gallic acid	ND	-	169.100 → 125.050	-
Chlorogenic acid	ND	-	353.100 → 191.200	-
Resveratrol	ND	-	227.000 → 143.000	-
Quercetin	ND	-	301.040 → 151.100	-
Genistein	ND	-	269.000 → 133.000	-

ND—not detected; LOQ—limit of quantification. Concentrations normalized to dry extract were calculated using an extract concentration of 10 mg/mL. The normalized LOQ was 0.005 mg/g dry extract. Values are presented as mean ± SD of three technical determinations performed using the same analytical solution prepared from a single extract batch. The reported SD therefore reflects within-sample analytical repeatability and not variability between independently prepared extraction batches. * Rutin was detected but could not be reliably quantified because its concentration was below the LOQ of 0.05 µg/mL. Where chromatographic or mass-spectrometric discrimination between structural isomers was not possible, concentrations are reported collectively for the corresponding analyte group.

## Data Availability

All the data obtained are included in the present article. Further inquiries can be directed to the corresponding author.

## Abbreviations

3D	Three-dimensional
AO	Acridine orange
AOC	Antioxidant capacity
DMSO	Dimethyl sulfoxide
DPPH	2,2-Diphenyl-1-picrylhydrazyl
ESI	Electrospray ionization
FBS	Fetal bovine serum
GAE	Gallic acid equivalents
IC_50_	Half-maximal inhibitory concentration
JC-1	5,5′,6,6′-Tetrachloro-1,1′,3,3′-tetraethylbenzimidazolylcarbocyanine iodide
Lb	Lemon balm (*Melissa officinalis*)
LOD	Limit of detection
LOQ	Limit of quantification
MS/MS	Tandem mass spectrometry
MTT	3-(4,5-Dimethylthiazol-2-yl)-2,5-diphenyltetrazolium bromide
ND	Not detected
NRU	Neutral red uptake
NSCLC	Non-small-cell lung cancer
PBS	Phosphate-buffered saline
PI	Propidium iodide
SD	Standard deviation
SLS	Sodium lauryl sulfate
TPC	Total phenolic content
UHPLC-MS/MS	Ultra-high-performance liquid chromatography–tandem mass spectrometry
UV-Vis	Ultraviolet–visible
ΔΨm	Mitochondrial membrane potential
